# Three Pairs of New Isopentenyl Dibenzo[*b*,*e*]oxepinone Enantiomers from *Talaromyces flavus,* a Wetland Soil-Derived Fungus

**DOI:** 10.3390/molecules21091184

**Published:** 2016-09-07

**Authors:** Tian-Yu Sun, Run-Qiao Kuang, Guo-Dong Chen, Sheng-Ying Qin, Chuan-Xi Wang, Dan Hu, Bing Wu, Xing-Zhong Liu, Xin-Sheng Yao, Hao Gao

**Affiliations:** 1 Institute of Traditional Chinese Medicine and Natural Products, College of Pharmacy, Jinan University, Guangzhou 510632, China; sty4048588@163.com (T.-Y.S.); bridgekuang@163.com (R.-Q.K.); wcxjnu@163.com (C.-X.W.); thudan@jnu.edu.cn (D.H.); tyaoxs@jnu.edu.cn (X.-S.Y.); 2 Clinical Experimental Center, First Affiliated Hospital of Jinan University, Guangzhou 510632, China; qinshengying78@163.com; 3 State Key Laboratory of Mycology, Institute of Microbiology, Chinese Academy of Sciences, Beijing 100190, China; wubing@im.ac.cn (B.W.); liuxz@sun.im.ac.cn (X.-Z.L.)

**Keywords:** isopentenyl dibenzo[*b*,*e*]oxepinone, wetland soil-derived fungus, *Talaromyces flavus*, arugosins K–N

## Abstract

Three pairs of new isopentenyl dibenzo[*b*,*e*]oxepinone enantiomers, (+)-(5*S*)-arugosin K (**1a**), (−)-(5*R*)-arugosin K (**1b**), (+)-(5*S*)-arugosin L (**2a**), (−)-(5*R*)-arugosin L (**2b**), (+)-(5*S*)-arugosin M (**3a**), (−)-(5*R*)-arugosin M (**3b**), and a new isopentenyl dibenzo[*b*,*e*]oxepinone, arugosin N (**4**), were isolated from a wetland soil-derived fungus *Talaromyces flavus*, along with two known biosynthetically-related compounds **5** and **6**. Among them, arugosin N (**4**) and 1,6,10-trihydroxy-8-methyl-2-(3-methyl-2-butenyl)-dibenz[*b*,*e*]oxepin-11(6*H*)-one (CAS: 160585-91-1, **5**) were obtained as the tautomeric mixtures. The structures of isolated compounds were determined by detailed spectroscopic analysis. In addition, the absolute configurations of these three pairs of new enantiomers were determined by quantum chemical ECD calculations.

## 1. Introduction

Isopentenyl dibenzo[*b*,*e*]oxepinones possess a dibenzo[*b*,*e*]oxepin-11(6*H*)-one skeleton (intact 6-7-6 tricyclic system) with isopentenyl substitution. Up to now, about nine isopentenyl dibenzo[*b*,*e*]oxepinones had been reported from fungi in Nature, including arugosins A–D from *Aspergillus rugulosus* [1,2] and *A. variecolor* [3], arugosin G from *Emericella nidulans* var. *acristata* [4], pestalachloride B from *Pestalotiopsis adusta* [5], cephalanones D–E from *Graphiopsis chlorocephala* [6], and 1,6,10-trihydroxy-8-methyl-2-(3-methyl-2-butenyl)-dibenz[*b*,*e*]oxepin-11(6*H*)-one (CAS: 160585-91-1, **5**) from *Penicilium* sp. [7].

**Figure 1:**
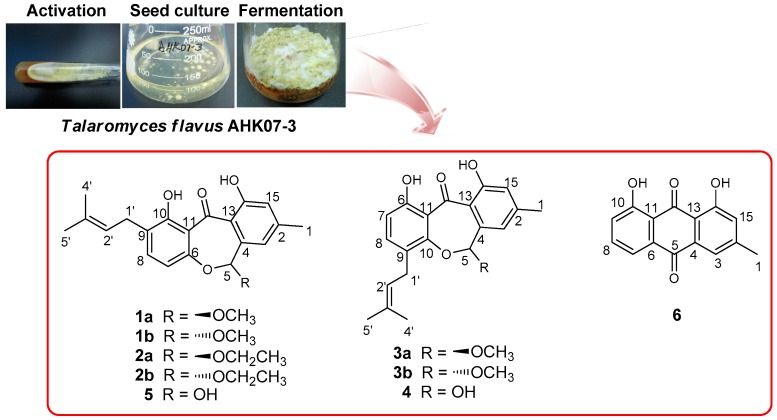
Production and chemical structures of **1**–**6**.

*Talaromyces flavus*, the commonest species of the genus *Talaromyces*, has been reported to produce a series of bioactive compounds [8,9]. In our search for bioactive secondary metabolites from wetland fungi [10,11,12,13,14,15], several interesting molecules also had been isolated from this species [12,13,14,15]. At present, a chemical investigation of metabolites from *Talaromyces flavus* AHK07-3 was carried out, which led to the isolation of three pairs of new isopentenyl dibenzo[*b*,*e*]oxepinone enantiomers ((+)-(5*S*)-arugosin K (**1a**), (−)-(5*R*)-arugosin K (**1b**), (+)-(5*S*)-arugosin L (**2a**), (−)-(5*R*)-arugosin L (**2b**), (+)-(5*S*)-arugosin M (**3a**), (−)-(5*R*)-arugosin M (**3b**)), a new isopentenyl dibenzo[*b*,*e*]oxepinone, arugosin N (**4**), and two known compounds 1,6,10-trihydroxy-8-methyl-2-(3-methyl-2-butenyl)-dibenz[*b*,*e*]oxepin-11(6*H*)-one (**5**) and chrysophanol (**6**) (Figure 1). The absolute configurations of the three pairs of enantiomers were determined by quantum chemical ECD calculations. In addition, arugosin N (**4**) and 1,6,10-trihydroxy-8-methyl-2-(3-methyl-2-butenyl)-dibenz[*b*,*e*]oxepin-11(6*H*)-one (**5**) were a pair of tautomers, and they were isolated as mixtures. Detail of the isolation and structural elucidations of compounds **1**–**6** are presented herein.

## 2. Results and Discussion

**Figure 2:**
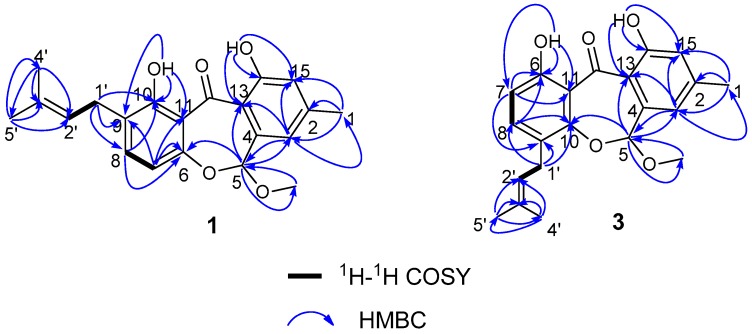
Key ^1^H-^1^H COSY and HMBC correlations of **1** and **3**.

Compound **1** was obtained as a yellow solid. The quasi-molecular ion at *m*/*z* 355.1550 [M + H]^+^ obtained by HRESIMS indicated the molecular formula of **1** was C_21_H_22_O_5_ with 11 degrees of unsaturation. The ^13^C-NMR spectrum combined with the DEPT-135 spectrum showed 21 signals (Table 1), assigned to ten sp^2^ quaternary carbons [including one ketone carbonyl (δ_C_ 197.5), nine aromatic/olefinic carbons (δ_C_ 162.8, 162.4, 154.5, 147.1, 138.5, 133.2, 124.8, 116.8, and 113.6), five sp^2^ methine carbons (δ_C_ 137.6, 121.8, 119.5, 116.8, and 109.3), one sp^3^ oxygenated methine carbon (δ_C_ 103.4), one sp^3^ methylene carbon (δ_C_ 27.8), and four methyl carbons (δ_C_ 56.9, 25.8, 21.8, and 17.8). In the ^1^H-NMR spectrum of **1** (Table 1), the characteristic signals of two exchangeable protons (δ_H_ 13.62 (1H, s) and 11.46 (1H, s)), five aromatic or olefinic protons (δ_H_ 7.33 (1H, d, *J* = 8.3 Hz), 6.94 (1H, s), 6.87 (1H, s), 6.58 (1H, d, *J* = 8.3 Hz), and 5.33 (1H, br t, *J* = 7.3 Hz), one sp^3^ oxygenated methine proton (δ_H_ 5.64 (1H, s)), one sp^3^ methylene (δ_H_ 3.34 (2H, d, *J* = 7.3 Hz)), and four methyl protons (δ_H_ 3.57 (3H, s), 2.37 (3H, s), 1.77 (3H, br s), and 1.73 (3H, br s)) were observed. The non-exchangeable proton resonances were associated with the directly attached carbon atoms in the HSQC experiment (Table 1). The analysis of the ^1^H-^1^H COSY experiment and the coupling values of the protons indicated the presence of two subunits (C-7–C-8 and C-1′–C-2′) as shown in Figure 2. Combined with the analysis of the ^1^H-^1^H COSY experiment and molecular formula, the HMBC correlations from H_3_-1 to C-2/C-3/C-15, from H-3 to C-1/C-5/C-13/C-15, from H-5 to C-3/C-6/C-13/5-OCH_3_, from H-7 to C-6/C-9/C-11, from H-8 to C-6/C-10, from H_2_-1′ to C-8/C-9/C-10, from H_3_-4′ to C-2′/C-3′/C-5′, from H_3_-5′ to C-2′/C-3′/C-4′, from 5-OCH_3_ to C-5, from 10-OH to C-9/C-10/C-11, and from 14-OH to C-13/C-14/C-15 revealed the planer structure of **1** (Figure 2). On the basis of the above analyses, the planar structure of **1** (Figure 2) was established, and it was named arugosin K. The assignments of all proton and carbon resonances are provided in Table 1.

**Figure 3:**
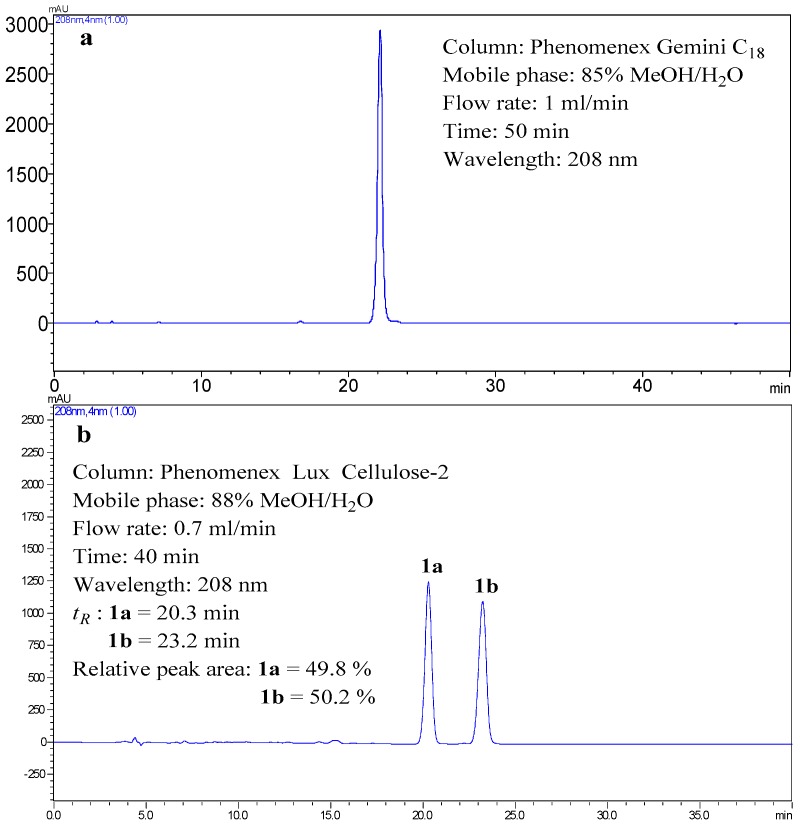
HPLC analysis of **1**. **a**: the analysis of **1** on routine ODS HPLC; **b**: the analysis of **1** on chiral HPLC.

The specific rotation value of **1** was close to zero, indicating that **1** was a racemic mixture. Compound **1** displayed two peaks in a Phenomenex Lux Cellulose-2 chiral column (Figure 3), which confirmed the above deduction. Therefore, (+)-**1a** (*t*_R_ = 20.3 min) and (−)-**1b** (*t*_R_ = 23.2 min) were isolated from **1** by a Phenomenex Lux Cellulose-2 chiral column using CH_3_OH/H_2_O (88/12, *v*/*v*) as eluent at a flow rate of 0.7 mL/min. The ECD spectra and ${\left[\alpha \right]}_{D}$ values of **1a** (${\left[\alpha \right]}_{D}^{27}$ +27.8 (*c* 0.5, CHCl_3_)) and **1b** (${\left[\alpha \right]}_{D}^{27}$ −27.0 (*c* 0.5, CHCl_3_)) showed their enantiomeric relationship.

**Figure 4:**
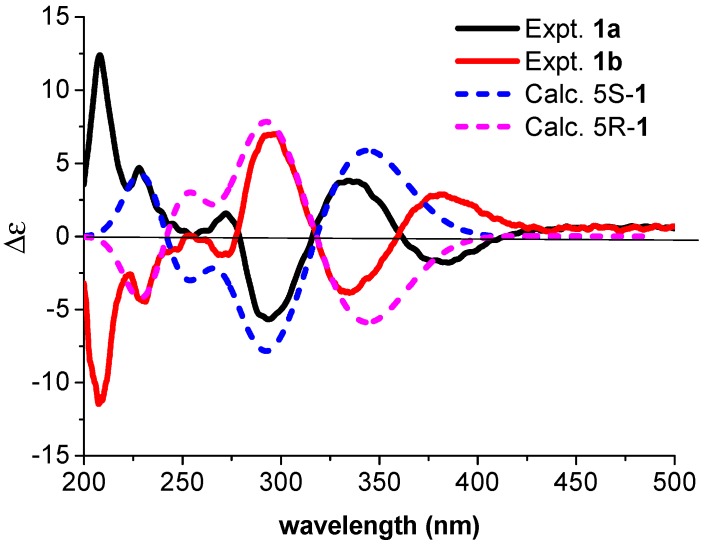
The experimental ECD spectra of **1a** and **1b** and calculated ECD spectra for (5*S*)-**1** and (5*R*)-**1** (UV correction = −10 nm, band width σ = 0.3 eV).

The absolute configurations of **1a** and **1b** were determined by quantum chemical ECD calculations at the APFD/6-311++G(2d,p) level. The predicted ECD curves of (5*S*)-**1** and (5*R*)-**1** (Figure 4) were in good accordance with the experimental ECD for **1a** and **1b**, respectively. Therefore, the absolute configurations of **1a** and **1b** were identified as 5*S* and 5*R*, respectively.

Compound **2** was obtained as a yellow solid. The quasi-molecular ion at *m*/*z* 369.1695 [M + H]^+^ by HRESIMS indicated the molecular formula of **2** was C_22_H_24_O_5_ with 11 degrees of unsaturation. Except for the loss of an oxygenated methyl at δ_C_ 56.9 (5-OCH_3_) and the appearance of an additional oxygenated methylene at δ_C_ 65.3 (C-1′′) and methyl at δ_C_ 14.8 (C-2′′), the NMR data of **2** (Table 1) were similar to those of **1** (Table 1), which indicated that the 5-OCH_3_ in **1** was replaced by 5-OCH_2_CH_3_ in **2**. This deduction was supported by the ^1^H-^1^H COSY correlations of Ha-1′′/Hb-1′′ and H_3_-2′′ and the key HMBC correlations from H-5 to C-1′′, from Ha-1′′/Hb-1′′ to C-5/C-2′′, and from H_3_-2′′ to C-1′′. On the basis of the exhaustive analysis of 2D NMR data (^1^H-^1^H COSY, HSQC, and HMBC) (Table S2, Supplementary Materials), the planar structure of **2**, named arugosin L, was established (Figure 1).

**Figure 5:**
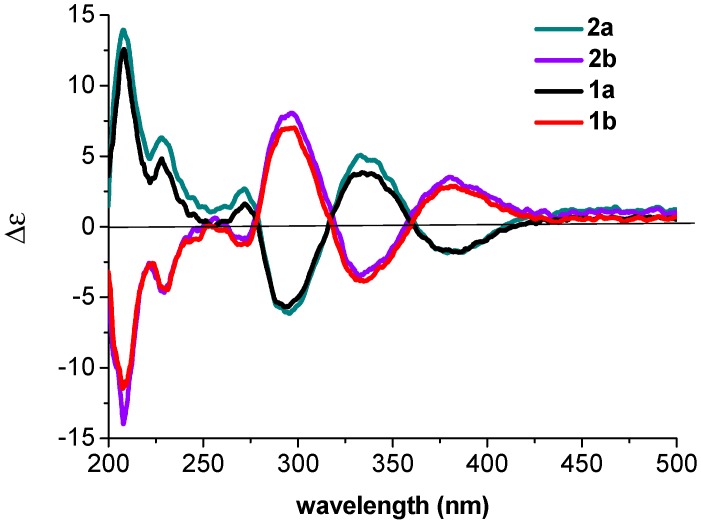
The experimental ECD spectra of **2a**, **2b**, **1a**, and **1b**.

Since **1** and **2** coexist in the same strain, **2** was also a racemic mixture, and it displayed two peaks in a Phenomenex Lux Cellulose-2 chiral column (Figure S1, Supplementary Materials). Therefore, (+)-**2a** (*t*_R_ = 22.6 min) and (−)-**2b** (*t*_R_ = 26.6 min) were isolated from **2** by a Phenomenex Lux Cellulose-2 chiral column using CH_3_OH/H_2_O (88/12, *v*/*v*) as eluent at a flow rate of 0.7 mL/min. The ECD spectra and ${\left[\alpha \right]}_{D}$ values of **2a** (${\left[\alpha \right]}_{D}^{27}$ +27.4 (*c* 0.5, CHCl_3_)) and **2b** (${\left[\alpha \right]}_{D}^{27}$ −26.8 (*c* 0.5, CHCl_3_)) showed their enantiomeric relationship. Since the ECD data of 2a were similar to that of **1a** (Figure 5), the absolute configuration of **2a** was determined as 5*S* (Figure 5). The similar observation was found between **2b** and **1b**. Therefore, the absolute configurations of **2a** and **2b** were assigned as 5*S* and 5*R*, respectively.

Compound **3** was obtained as a yellow solid. The quasi-molecular ion at *m*/*z* 355.1541 [M + H]^+^ by HRESIMS indicated the molecular formula of **3** was C_21_H_22_O_5_ with 11 degrees of unsaturation, which indicated that **3** was the isomer of **1**. Furthermore, the NMR data of **3** (Table 1) were similar to those of **1**, which suggested that **3** and **1** had the similar skeleton. The analysis of the ^1^H-^1^H COSY experiment and the coupling values of the protons indicated the presence of two subunits (C-7–C-8 and C-1′–C-2′) as shown in Figure 2. Combined with the analysis of the ^1^H–^1^H COSY experiment and molecular formula, the HMBC correlations (Figure 2) from H_3_-1 to C-2/C-3/C-15, from H-3 to C-1/C-5/C-13/C-15, from H-5 to C-3/C-10/C-13/5-OCH_3_, from H-7 to C-6/C-9/C-11, from H-8 to C-6/C-10, from Ha-1′/Hb-1′ to C-8/C-9/C-10, from H_3_-4′ to C-2′/C-3′/C-5′, from H_3_-5′ to C-2′/C-3′/C-4′, from 5-OCH_3_ to C-5, from 6-OH to C-6/C-7/C-11, and from 14-OH to C-13/C-14/C-15 confirmed the planar structure of **3**, which is named arugosin M.

**Figure 6:**
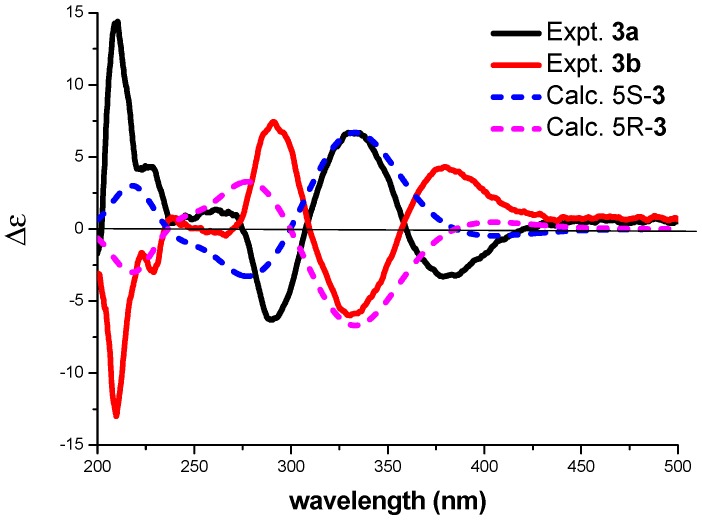
The experimental ECD spectra of **3a** and **3b** and calculated ECD spectra for (5*S*)-**3** and (5*R*)-**3** (UV correction = −20 nm, band width σ = 0.3 eV).

Since **1** and **3** coexist in the same strain, **3** was also a racemic mixture, and it displayed two peaks in a Phenomenex Lux Cellulose-2 chiral column (Figure S2, Supplementary Materials). Therefore, (+)-**3a** (*t*_R_ = 20.4 min) and (−)-**3b** (*t*_R_ = 22.1 min) were isolated from **3** by a Phenomenex Lux Cellulose-2 chiral column using CH_3_OH/H_2_O (88/12, *v*/*v*) as eluent at a flow rate of 0.7 mL/min. The ECD spectra and ${\left[\alpha \right]}_{D}$ values of **3a** (${\left[\alpha \right]}_{D}^{27}$ +28.4 (*c* 0.5, CHCl_3_)) and **3b** (${\left[\alpha \right]}_{D}^{27}$ −27.6 (*c* 0.5, CHCl_3_)) showed their enantiomeric relationship. The absolute configurations of **3a** and **3b** were determined by quantum chemical ECD calculations at the APFD/6-311++g(2d,p) level. The predicted ECD curves of (5*S*)-**3** and (5*R*)-**3** were in good accordance with the experimental ECD for **3a** and **3b**, respectively (Figure 6). Therefore, the absolute configurations of **3a** and **3b** were identified as 5*S* and 5*R*, respectively.

Compounds **4** and **5** were obtained as mixtures of yellow solids. The quasi-molecular ion at *m*/*z* 363.1213 [M + Na]^+^ by HRESIMS indicated the molecular formulas of **4** and **5** were C_20_H_20_O_5_, with 11 degrees of unsaturation. The NMR data of the mixtures presented two sets of signals, assigned to **4** and **5** respectively (Table 2). The molecular formula of **4** was fourteen atomic mass units less than **3**. Except for the loss of an oxygenated methyl at δ_C_ 57.3 (5-OCH_3_), the ^1^H NMR and ^13^C-NMR data of **4** (Table 2) were similar to those of **3** (Table 1), which indicated that the 5-OCH_3_ in **3** was replaced by the hydroxyl in **4**. On the basis of the exhaustive analysis of 2D NMR data (^1^H-^1^H COSY, HSQC, and HMBC) (Table S4, Supplementary Materials), the planar structure of **4** was established, named arugosin N.

The molecular formula of **5** was fourteen atomic mass units less than **1**. Except for the loss of an oxygenated methyl at δ_C_ 56.9 (5-OCH_3_), the ^1^H-NMR and ^13^C-NMR data of **5** (Table 2) were similar to those of **1** (Table 1), which indicated that the 5-OCH_3_ in **1** was replaced by the hydroxyl in **5**. On the basis of the exhaustive analysis of 2D NMR data (^1^H-^1^H COSY, HSQC, and HMBC) (Table S5, Supplementary Materials), the planar structure of **5** was established as the known compound, 1,6,10-trihydroxy-8-methyl-2-(3-methyl-2-butenyl)-dibenz[*b*,*e*]oxepin-11(6*H*)-one [7].

**Figure 7:**
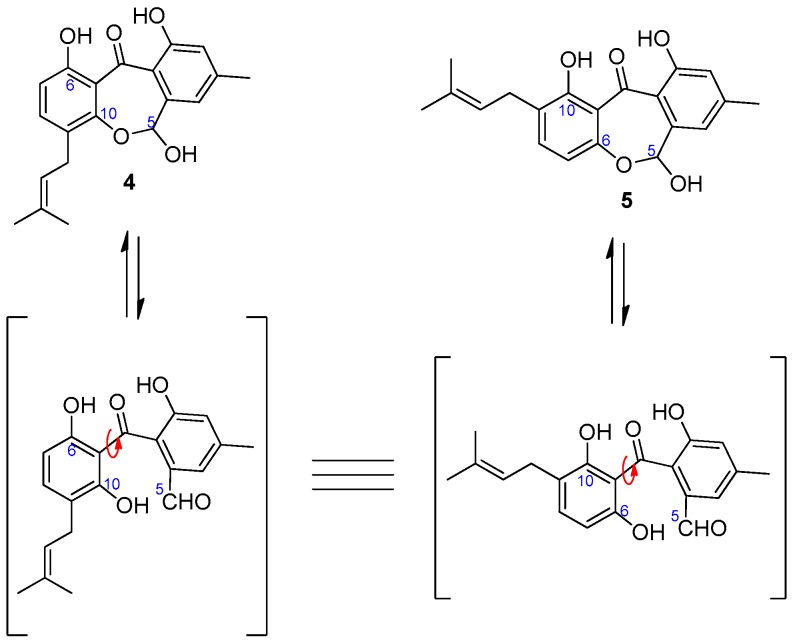
The interconversion of **4** and **5**.

The C-5 positions of **4** and **5** were hemiacetal linkage, which were unstable and easy to split. Therefore, **4** and **5** can self-interconvert (Figure 7) and exist as inseparable tautomeric mixtures in Nature.

Compound **6** was obtained as a yellow solid. The ESI-MS (positive): *m*/*z* 255 [M + H]^+^; ESI-MS (negative): *m*/*z* 253 [M − H]^−^ indicated the molecular weight was 254. It was identified as chrysophanol by comparing its data (^1^H- and ^13^C-NMR, MS) with literature values [16].

**Scheme 1:**
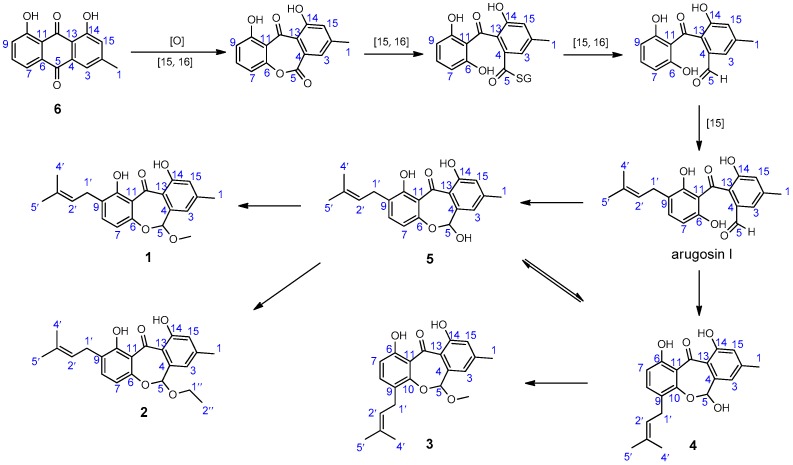
The plausible biosynthetic pathways of **1**–**6**.

Based on the structural features of compounds **1**–**6** and literature investigation, a plausible biosynthetic pathway of them was proposed (Scheme 1). Chrysophanol (**6**) undergoes oxidization [17,18], ring-cleavage [17,18], reduction [17,18], and isoprentenylation to yield arugosin I [17], which may be the precursor of arugosin N (**4**) and 1,6,10-trihydroxy-8-methyl-2-(3-methyl-2-butenyl)-dibenz[*b,e*]oxepin-11(6*H*)-one (**5**). Then, arugosin K (**1**) and arugosin L (**2**) may originate from **5** by methylation and ethylation, respectively. Arugosin M (**3**) may originate from **4** by methylation.

## 3. Materials and Methods

### 3.1. Chemicals

Methanol (MeOH) was purchased from Yuwang Industrial Co. Ltd. (Yucheng, China). Cyclohexane, ethyl acetate (EtOAc), and petroleum ether (PE) were purchased from Tianjin Fuyu Fine Chemical Co. Ltd. (Tianjin, China).

### 3.2. General Experimental Procedures

Optical rotations were measured on a P1020 digital polarimeter (Jasco International Co. Ltd., Tokyo, Japan). UV data were recorded using a Jasco V-550 UV/Vis spectrometer. IR data were recorded on a Jasco FT/IR-480 plus spectrometer. ECD spectrum was recorded on a Jasco J-810 spectrophotometer using MeOH as the solvent. The ESI-MS spectra were recorded on a Finnigan LCQ Advantage MAX mass spectrometer (Thermo Fisher Scientific, Inc., Waltham, MA, USA). The HR-ESI-MS spectra were obtained on a Micromass Q-TOF mass spectrometer (Waters Corporation, Milford, MA, USA). The NMR spectra were measured with Bruker AV-400 and Bruker AV-600 spectrometers (Bruker BioSpin Group, Faellanden, Switzerland) using the solvent signals (CDCl_3_: δ_H_ 7.26/δ_C_ 77.0) as internal standard. The analytical HPLC was performed on a Dionex HPLC system equipped with an Ultimate 3000 pump, an Ultimate 3000 diode array detector (DAD), an Ultimate 3000 Column Compartment, an Ultimate 3000 autosampler (Thermo Fisher Scientific, Inc.) using a Gemini C_18_ column (4.6 mm × 250 mm, 5 μm) (Phenomenex Inc., Los Angeles, CA, USA). The semi-preparative HPLC was performed on a Shimadzu LC-6-AD Liquid Chromatography with an SPD-20A Detector using a Phenomenex Gemini C_18_ column (Phenomenex Inc.) (10.0 mm × 250 mm, 5 μm). The chiral HPLC was performed on a Shimadzu LC-6-AD Liquid Chromatography with an SPD-20A Detector using a Phenomenex Lux Cellulose-2 chiral column (4.6 mm × 250 mm, 3 μm).

### 3.3. Fungus Material

The strain AHK07-3 was isolated from the soil, collected at the wetland of Ahongkou, Sinkiang Province, China. The strain was identified as *Talaromyces flavus* based on the morphological characters and gene sequence analysis. The ribosomal internal transcribed spacer (ITS) and the 5.8S rRNA gene sequences (ITS1-5.8S-ITS2) of the strain have been deposited at GenBank as KX011167. The fungus was cultured on slants of potato dextrose agar at 25 °C for 5 days. Agar plugs were used to inoculate four Erlenmeyer flasks (250 mL), each containing 100 mL of potato dextrose broth. Four flasks of the inoculated media were incubated at 25 °C on a rotary shaker at 200 rpm for 5 days to prepare the seed culture. Fermentation was carried out in 20 Erlenmeyer flasks (500 mL), each containing 70 g of rice. Distilled H_2_O (105 mL) was added to each flask, and the rice was soaked overnight before autoclaving at 120 °C for 30 min. After cooling to room temperature, each flask was inoculated with 5.0 mL of the seed culture containing mycelia and incubated at room temperature for 50 days.

### 3.4. Extraction and Isolation

The culture was extracted with EtOAc (3 × 5000 mL). Removal of EtOAc under reduced pressure yielded a crude extract (19.7 g) that was dissolved in 90% *v*/*v* aqueous MeOH (400 mL). The methanolic solution was further extracted with cyclohexane (400 mL) to afford a cyclohexane fraction C (4.6 g). Fraction C was subjected to silica gel (200–300 mesh) column chromatography initially eluting with cyclohexane (500 mL) and then with MeOH (500 mL) to afford subfractions CC and CM. Subfraction CC (3.3 g) was further separated by open silica gel column chromatography eluting with cyclohexane–EtOAc (100:0 (150 mL), 99:1 (150 mL), 98:2 (150 mL), 97:3 (150 mL), 96:4 (150 mL), 95:5 (150 mL), 94:6 (150 mL), 93:7 (150 mL), 92:8 (150 mL), 91:9 (150 mL), 90:10 (150 mL), 50:50 (150 mL), and 0:100 (150 mL), *v*/*v*) to yield four sub-subfractions (CC1 to CC4). Sub-subfraction CC2 (2.8 g) was subjected to open silica gel column chromatography eluting with PE (petroleum ether)–EtOAc (100:0 (100 mL), 99:1 (100 mL), 98:2 (100 mL), 97:3 (100 mL), 96:4 (100 mL), 95:5 (100 mL), 94:6 (100 mL), 93:7 (100 mL), 92:8 (100 mL), 91:9 (100 mL), 90:10 (100 mL), 50:50 (100 mL) and 0:100 (100 mL), *v*/*v*) to yield four sub-subfractions (CC2a to CC2d). Sub-subfraction CC2b (1.1 g) was isolated by semi-preparative HPLC purification using MeOH–H_2_O (88:12, *v*/*v*) at a flow rate of 3 mL/min to yield **1** (617.4 mg), **2** (2.6 mg), and **3** (24.7 mg). Sub-subfraction CC2c (0.6 g) was purified by semi-preparative HPLC purification using MeOH–H_2_O (90:10, *v*/*v*) at a flow rate of 3 mL/min to yield **6** (1.1 mg). Sub-subfraction CC4 (220.1 mg) was isolated by semi-preparative HPLC purification using MeOH–H_2_O (80:20, *v*/*v*) at a flow rate of 3 mL/min to yield the mixtures of **4** and **5** (12.5 mg). Enantioseparation of **1**–**3** was carried out on a Phenomenex Lux Cellulose-2 chiral column using CH_3_OH/H_2_O (88/12, *v*/*v*) as mobile phase.

### 3.5. Spectroscopic Data of Isolated Compounds

*(±)-Arugosin K* (**1**): yellow solid; UV (MeOH) λ_max_ (log ε) 205 (3.75), 221 (3.63), 304 (3.35), 359 (3.29) nm; IR (KBr) ν_max_ 3438, 2920, 1708, 1619, 1590, 1421, 1206, 1052 cm^−1^; ESI-MS (positive): *m*/*z* 355 [M + H]^+^, ESI-MS (negative): *m*/*z* 353 [M − H]^−^; HRESIMS (positive) *m/z* 355.1550 [M + H]^+^ (calcd. for C_21_H_23_O_5_, 355.1545); ^1^H- and ^13^C-NMR data see Table 1.

*(+)-S-Arugosin K* (**1a**): ${\left[\alpha \right]}_{D}^{27}$ +27.8 (*c* 0.5, CHCl_3_); ECD (*c* 8.5 × 10^−5^ M, MeOH) λ_max_ (Δε) 208 (+12.50), 294 (−5.71), 334 (+3.76), 382 (−1.77) nm.

*(−)-R-Arugosin K* (**1b**): ${\left[\alpha \right]}_{D}^{27}$ −27.0 (*c* 0.5, CHCl_3_); ECD (*c* 8.5 × 10^−5^ M, MeOH) λ_max_ (Δε) 208 (−11.38), 295 (+6.96), 334 (−3.88), 382 (+2.82) nm.

*(±)-Arugosin L* (**2**): yellow solid; UV (MeOH) λ_max_ (log ε) 206 (3.70), 222 (3.57), 304 (3.30), 359 (3.23) nm; IR (KBr) ν_max_ 3440, 2916, 1624, 1590, 1422, 1206, 1057 cm^−1^; ESI-MS (positive): *m*/*z* 369 [M + H]^+^; ESI-MS (negative): *m*/*z* 367 [M − H]^−^; HRESIMS (positive) *m*/*z* 369.1695 [M + H] ^+^ (calcd. for C_22_H_25_O_5_, 369.1702); ^1^H- and ^13^C-NMR data see Table 1.

*(+)-S-Arugosin L* (**2a**): ${\left[\alpha \right]}_{D}^{27}$ +27.4 (*c* 0.5, CHCl_3_); ECD (*c* 6.8 × 10^−5^ M, MeOH) λ_max_ (Δε) 208 (+13.90), 295 (−6.07), 335 (+5.07), 382 (−1.91) nm.

*(−)-R-Arugosin L* (**2b**): ${\left[\alpha \right]}_{D}^{27}$ −26.8 (*c* 0.5, CHCl_3_); ECD (*c* 6.8 × 10^−5^ M, MeOH) λ_max_ (Δε) 208 (−13.79), 295 (+7.98), 335 (−3.44), 382 (+3.40) nm.

*(±)-Arugosin M* (**3**): yellow solid; UV (MeOH) λ_max_ (log ε) 206 (3.85), 221 (3.73), 299 (3.42), 357 (3.36) nm; IR (KBr) ν_max_ 3441, 2913, 1620, 1464, 1202, 1050 cm^−1^; ESI-MS (positive): *m*/*z* 355 [M + H]^+^, ESI-MS (negative): *m*/*z* 353 [M − H]^−^; HRESIMS (positive) *m/z* 355.1541 [M + H]^+^ (calcd. for C_21_H_23_O_5_, 355.1545); ^1^H- and ^13^C-NMR data see Table 1.

*(+)-S-Arugosin M* (**3a**): ${\left[\alpha \right]}_{D}^{27}$ +28.4 (*c* 0.5, CHCl_3_); ECD (*c* 8.5 × 10^−5^ M, MeOH) λ_max_ (Δε) 209 (+14.24), 290 (−6.29), 334 (+6.67), 381 (−3.30) nm.

*(−)-R-Arugosin M* (**3b**): ${\left[\alpha \right]}_{D}^{27}$ −27.6 (*c* 0.5, CHCl_3_); ECD (*c* 8.5 × 10^−5^ M, MeOH) λ_max_ (Δε) 209 (−12.84), 292 (+7.26), 334 (−5.93), 381 (+4.27) nm.

*Arugosin N* (**4**): 1,6,10-trihydroxy-8-methyl-2-(3-methyl-2-butenyl)-dibenz[*b*,*e*]oxepin-11(6*H*)-one (**5**): yellow solid; UV (MeOH) λ_max_ (log ε) 206 (3.75), 222 (3.65), 286 (3.29), 356 (3.16) nm; IR (KBr) ν_max_ 3443, 2917, 1628, 1589, 1420, 1353, 1173, 1052 cm^−1^; ESI-MS (positive): *m*/*z* 341 [M + H]^+^, *m*/*z* 363 [M + Na]^+^; ESI-MS (negative): *m*/*z* 339 [M − H]^−^; HRESIMS (positive) *m*/*z* 363.1213 [M + Na]^+^ (calcd. for C_20_H_20_O_5_Na, 363.1208); ^1^H- and ^13^C-NMR data see Table 2.

### 3.6. ECD Calculation

Before molecular initial 3D structures were generated with CORINA version 3.4 (Molecular Networks GmbH, Erlangen, Germany), the molecules of (5*S*)-**1**, (5*R*)-**1**, (5*S*)-**3**, and (5*R*)-**3** were converted into SMILES codes, respectively. Conformer databases were generated in CONFLEX version 7.0 (CONFLEX Corporation, Tokyo, Japan) using the MMFF94s force-field, with an energy window for acceptable conformers (ewindow) of 5 kcal·mol^−1^ above the ground state, a maximum number of conformations per molecule (maxconfs) of 100, and an RMSD cutoff (rmsd) of 0.5 Å. Then each conformer of the acceptable conformers was optimized with HF/6-31G(d) method in Gaussian09 [19]. Further optimization at the APFD/6-31G(d) level led the dihedral angles to be got. After that, eight lowest energy conformers of (5*S*)-**1** and (5*R*)-**1** (see Supplementary Materials Table S6 and Figure S3) were found out. Six lowest energy conformers of (5*S*)-**3** and (5*R*)-**3** (see Supplementary Materials Table S7 and Figure S4) were found out. The optimized conformers were taken for the ECD calculations, which were performed with Gaussian09 (APFD/6-311++G(2d,p)). The solvent effect was taken into account by the polarizable-conductor calculation model (IEFPCM, methanol as the solvent). Comparisons of the experimental and calculated spectra were done with the software SpecDis [20,21]. It was also used to apply a UV shift to the ECD spectra, Gaussian broadening of the excitations, and Boltzmann weighting of the spectra.

## 4. Conclusions

Up to now, about nine isopentenyl dibenzo[*b*,*e*]oxepinones with intact 6-7-6 tricyclic systems have been reported from the genera of *Aspergillus*, *Emericella*, *Pestalotiopsis*, *Graphiopsis*, and *Penicilium*. Except the absolute configuration of cephalanone D that had been verified by X-ray crystallography [6], the absolute configurations of the others had not been not investigated. In our study, the absolute configurations of the three pairs of enantiomers ((+)-(5*S*)-**1a**, (−)-(5*R*)-**1b**, (+)-(5*S*)-**2a**, (−)-(5*R*)-**2b**, (+)-(5*S*)-**3a**, (−)-(5*R*)-**3b**), isolated for the first time from the genus *Talaromyces*, were determined by quantum chemical ECD calculations, which is the first report for the absolute configuration of isopentenyl dibenzo[*b*,*e*]oxepinones established by ECD experiments. Furthermore, 1,6,10-trihydroxy-8-methyl-2-(3-methyl-2-butenyl)-dibenz[*b*,*e*]oxepin-11(6*H*)-one (**5**) is a known compound, but no NMR data was previously reported. The assignments of NMR data of **5** are thus provided for the first time.

## Figures and Tables

**Table 1:** The ^13^C- and ^1^H-NMR data of **1**–**3** (δ in ppm, *J* in Hz).

1 ^a^			2 ^b^			3 ^a^		
No.	δ_C_	δ_H_	No.	δ_C_	δ_H_^c^	No.	δ_C_	δ_H_^c^
1	21.8	2.37, s	1	21.9	2.38, s	1	21.9	2.39, s
2	147.1		2	147.2		2	147.1	
3	116.8	6.94, s	3	116.8	6.98, s	3	116.6	6.97, s
4	138.5		4	138.8		4	138.3	
5	103.4	5.64, s	5	102.0	5.74, s	5	103.9	5.71, s
6	154.5		6	154.8		6	163.5	
7	109.3	6.58, d (8.3)	7	109.3	6.55, d (8.3)	7	112.2	6.70, d (8.5)
8	137.6	7.33, d (8.3)	8	137.6	7.32, d (8.3)	8	138.4	7.35, d (8.5)
9	124.8		9	124.7		9	121.8	
10	162.8		10	162.7		10	153.2	
11	113.6		11	113.7		11	113.9	
12	197.5		12	197.6		12	197.5	
13	116.8		13	116.9		13	117.0	
14	162.4		14	162.3		14	161.9	
15	119.5	6.87, s	15	119.4	6.86, s	15	119.4	6.87, s
1′	27.8	3.34, d (7.3)	1′	27.8	3.35, dd (15.9, 7.4), a	1′	28.0	3.36, dd (15.6, 7.5), a
					3.31, dd (15.9, 7.4), b			3.30, dd (15.6, 7.5), b
2′	121.8	5.33, br t (7.3)	2′	121.8	5.32	2′	122.1	5.25
3′	133.2		3′	133.3		3′	133.2	
4′	25.8	1.77, br s	4′	25.8	1.76, br s	4′	25.7	1.73, br s
5′	17.8	1.73, br s	5′	17.8	1.73, br s	5′	17.8	1.71, br s
5-OCH_3_	56.9	3.57, s	1′′	65.3	3.96, dq (9.6, 7.1), a	5-OCH_3_	57.3	3.57, s
					3.66, dq (9.6, 7.1), b			
			2′′	14.8	1.26, t (7.1)			
10-OH		13.62, s	10-OH		13.63, s	6-OH		13.14, s
14-OH		11.46, s	14-OH		11.44, s	14-OH		11.22, s

^a^ The data recorded in CDCl_3_ (^1^H-NMR for 400 MHz, ^13^C-NMR for 100 MHz); ^b^ The data recorded in CDCl_3_ (^1^H-NMR for 600 MHz, ^13^C-NMR for 150 MHz); ^c^ Indiscernible signals owing to overlapping or having complex multiplicity are reported without designating multiplicity. The letters of a and b mean that these two potons on C-1′ are not chemical equivalence.

**Table 2:** The ^1^H-NMR and ^13^C-NMR data of **4** and **5** (δ in ppm, *J* in Hz).

4 ^a^			5 ^a^		
No.	δ_C_	δ_H_	No.	δ_C_	δ_H_
1	22.0	2.40, s	1	21.9	2.40, s
2	147.6		2	147.4	
3	116.0	7.07, s	3	116.2	7.04, s
4	139.7		4	139.3	
5	97.1	6.11, s	5	96.6	6.12, s
6	163.6		6	154.5	
7	112.9	6.71, d (8.4)	7	109.6	6.53, d (8.4)
8	138.5	7.34, d (8.4)	8	137.7	7.32, d (8.4)
9	122.4		9	125.1	
10	154.0		10	162.9	
11	114.6		11	113.5	
12	197.3		12	197.3	
13	116.5		13	116.7	
14	163.3		14	162.7	
15	119.8	6.89, s	15	119.7	6.89, s
1′	28.9	3.36, dd (15.4, 7.2), a	1′	27.8	3.33, d (7.5)
		3.22, dd (15.4, 7.2), b			
2′	123.1	5.24, br t (7.2)	2′	121.7	5.31, br t (7.5)
3′	133.0		3′	133.4	
4′	25.7	1.76, s	4′	25.8	1.76, s
5′	18.0	1.74, s	5′	17.8	1.72, s
6-OH		12.95, br s	10-OH		13.61, br s
14-OH		11.82, br s	14-OH		11.54, br s

^a^ The data recorded in CDCl_3_ (^1^H-NMR for 400 MHz, ^13^C-NMR for 100 MHz). The letters of a and b mean that these two potons on C-1′ are not chemical equivalence.

## Supplementary Materials

The following are available online at http://www.mdpi.com/1420-3049/21/9/1184/s1, The 1D and 2D NMR data of **1**–**5**, and quantum chemical ECD calculations of **1** and **3**.
