# Supplementary material for: Three Pairs of New Isopentenyl Dibenzo[b,e]oxepinone Enantiomers from Talaromyces flavus, a Wetland Soil-Derived Fungus
Source: Molecules. 2016 Sep 7;21(9):1184. doi: 10.3390/molecules21091184 (PMC6274342; doi:10.3390/molecules21091184)
Supplement: Supplementary file 1 [file molecules-21-01184-s001.pdf]

Supplementary Materials: Three Pairs of New Isopentenyl Dibenzo[*b*,*e*]oxepinone Enantiomers from *Talaromyces flavus*, a Wetland Soil-Derived Fungus

Tian-Yu Sun, Run-Qiao Kuang, Guo-Dong Chen, Sheng-Ying Qin, Chuan-Xi Wang, Dan Hu, Bing Wu, Xing-Zhong Liu, Xin-Sheng Yao, Hao Gao

Table S1. 1D and 2D data of **1** (^{1}H for 400 MHz, ^{13}C for 100 MHz in $CDCl_3$).

Position	δ_C, Type	δ_H (*J* in Hz)	^{1}H-^{1}H COSY	HMBC
1	21.8, CH_3	2.37, s		2, 3, 15
2	147.1, C			
3	116.8, CH	6.94, s		1, 5, 13, 15
4	138.5, C			
5	103.4, CH	5.64, s		3, 6, 13, O<u>C</u>H_3
6	154.5, C			
7	109.3, CH	6.58, d (8.3)	8	6, 9, 11
8	137.6, CH	7.33, d (8.3)	7	6, 10, 1′
9	124.8, C			
10	162.8, C			
11	113.6, C			
12	197.5, C			
13	116.8, C			
14	162.4, C			
15	119.5, CH	6.87, s		1, 3, 13, 14
1′	27.8, CH_2	3.34, d (7.3)	2′	8, 9, 10, 2′, 3′
2′	121.8, CH	5.33, br t (7.3)	1′, 4′, 5′	1′, 4′, 5′
3′	133.2, C			
4′	25.8, CH_3	1.77, br s	2′	2′, 3′, 5′
5′	17.8, CH_3	1.73, br s	2′	2′, 3′, 4′
O<u>C</u>H_3	56.9, CH_3	3.57, s		5
10-O<u>H</u>		13.62, s		9, 10, 11
14-O<u>H</u>		11.46, s		13, 14, 15

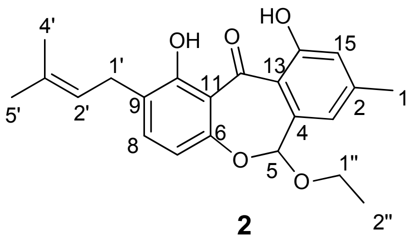

2

Table S2. 1D and 2D data of **2** (^1H for 600 MHz, ^{13}C for 150 MHz in $CDCl_3$).

Position	δC, Type	δH (*J* in Hz) [a]	^1H-^1H COSY	HMBC
1	21.9, CH_3	2.38, s		2, 3, 15
2	147.2, C			
3	116.8, CH	6.98, s		1, 5, 13, 15
4	138.8, C			
5	102.0, CH	5.74, s		3, 6, 13, 1″
6	154.8, C			
7	109.3, CH	6.55, d (8.3)	8	6, 9, 11
8	137.6, CH	7.32, d (8.3)	7	6, 10, 1′
9	124.7, C			
10	162.7, C			
11	113.7, C			
12	197.6, C			
13	116.9, C			
14	162.3, C			
15	119.4, CH	6.86, s		1, 3, 13, 14
1′	27.8, CH_2	3.35, dd (15.9, 7.4), a	1′b, 2′	8, 9, 10, 2′, 3′
		3.31, dd (15.9, 7.4), b	1′a, 2′	8, 9, 10, 2′, 3′
2′	121.8, CH	5.32	1′a, 1′b, 4′, 5′	1′, 4′, 5′
3′	133.3, C			
4′	25.8, CH_3	1.76, br s	2′	2′, 3′, 5′
5′	17.8, CH_3	1.73, br s	2′	2′, 3′, 4′
1″	65.3, CH_2	3.96, dq (9.6, 7.1), a	1″b, 2″	5, 2″
		3.66, dq (9.6, 7.1), b	1″a, 2″	5, 2″
2″	14.8, CH_3	1.26, t (7.1)	1″a, 1″b	1″
10-OH		13.63, s		9, 10, 11
14-OH		11.44, s		13, 14, 15

a: Indiscernible signals owing to overlapping or having complex multiplicity are reported without designating multiplicity.

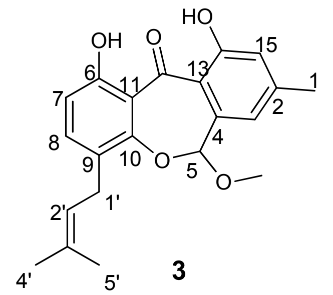

3

Table S3. 1D and 2D data of **3** (^{1}H for 400 MHz, ^{13}C for 100 MHz in $CDCl_3$).

Position	δ_C, Type	δ_H (*J* in Hz) [a]	^{1}H-^{1}H COSY	HMBC
1	21.9, CH_3	2.39, s		2, 3, 15
2	147.1, C			
3	116.6, CH	6.97, s		1, 5, 13, 15
4	138.3, C			
5	103.9, CH	5.71, s		3, 10, 13, O<u>C</u>H_3
6	163.5, C			
7	112.2, CH	6.70, d (8.5)	8	6, 9, 11
8	138.4, CH	7.35, d (8.5)	7	6, 10, 1′
9	121.8, C			
10	153.2, C			
11	113.9, C			
12	197.5, C			
13	117.0, C			
14	161.9, C			
15	119.4, CH	6.87, s		1, 3, 13, 14
1′	28.0, CH_2	3.36, dd (15.6, 7.5), a	1′b, 2′	8, 9, 10, 2′, 3′
		3.30, dd (15.6, 7.5), b	1′a, 2′	8, 9, 10, 2′, 3′
2′	122.1, CH	5.25	1′a, 1′b, 4′, 5′	1′, 4′, 5′
3′	133.2, C			
4′	25.7, CH_3	1.73, br s	2′	2′, 3′, 5′
5′	17.8, CH_3	1.71, br s	2′	2′, 3′, 4′
O<u>C</u>H_3	57.3, CH_3	3.57, s		5
6-O<u>H</u>		13.14, s		6, 7, 11
14-O<u>H</u>		11.22, s		13, 14, 15

a: Indiscernible signals owing to overlapping or having complex multiplicity are reported without designating multiplicity.

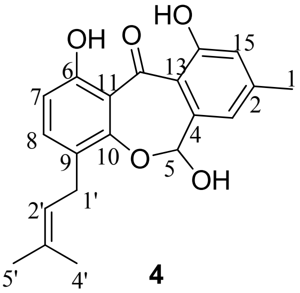

4

Table S4. 1D and 2D data of **4** (^1H for 400 MHz, ^{13}C for 100 MHz in $CDCl_3$).

Position	δC, Type	δH (*J* in Hz)	^1H-^1H COSY	HMBC
1	22.0, CH_3	2.40, s		2, 3, 15
2	147.6, C			
3	116.0, CH	7.07, s		1, 5, 13, 15
4	139.7, C			
5	97.1, CH	6.11, s		3, 10, 13
6	163.6, C			
7	112.9, CH	6.71, d (8.4)	8	9, 11
8	138.5, CH	7.34, d (8.4)	7	6, 10, 1′
9	122.4, C			
10	154.0, C			
11	114.6, C			
12	197.3, C			
13	116.5, C			
14	163.3, C			
15	119.8, CH	6.89, s		1, 3, 13
1′	28.9, CH_2	3.36, dd (15.4, 7.2), a	1′b, 2′	8, 9, 2′, 3′
		3.22, dd (15.4, 7.2), b	1′a, 2′	
2′	123.1, CH	5.24, br t (7.2)	1′a, 1′b, 4′,5′	5′
3′	133.0, C			
4′	25.7, CH_3	1.76, s	2′	2′, 3′, 5′
5′	18.0, CH_3	1.74, s	2′	2′, 3′, 4′
6-O<u>H</u>		12.95, br s		6, 7, 11
14-O<u>H</u>		11.82, br s		13, 14, 15

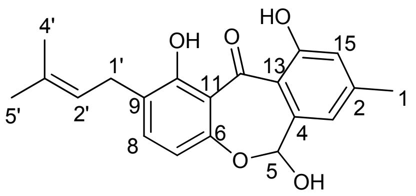

5

Table S5. 1D and 2D data of **5** (^{1}H for 400 MHz, ^{13}C for 100 MHz in $CDCl_3$).

Position	δ_C, Type	δ_H (*J* in Hz)	^{1}H-^{1}H COSY	HMBC
1	21.9, CH_3	2.40, s		2, 3, 15
2	147.4, C			
3	116.2, CH	7.04, s		1, 5, 13, 15
4	139.3, C			
5	96.6, CH	6.12, s		3, 6, 13
6	154.5, C			
7	109.6, CH	6.53, d (8.4)	8	6, 9, 11
8	137.7, CH	7.32, d (8.4)	7	6, 10, 1′
9	125.1, C			
10	162.9, C			
11	113.5, C			
12	197.3, C			
13	116.7, C			
14	162.7, C			
15	119.7, CH	6.89, s		1, 3, 13
1′	27.8, CH_2	3.33, d (7.5)	2′	8, 9, 10, 2′, 3′
2′	121.7, CH	5.31, br t (7.5)	1′, 4′, 5′	4′, 5′
3′	133.4, C			
4′	25.8, CH_3	1.76, s	2′	2′, 3′, 5′
5′	17.8, CH_3	1.72, s	2′	2′, 3′, 4′
10-O<u>H</u>		13.61, br s		9, 10, 11
14-O<u>H</u>		11.54, br s		13, 14, 15

HPLC Analyses of 2 and 3

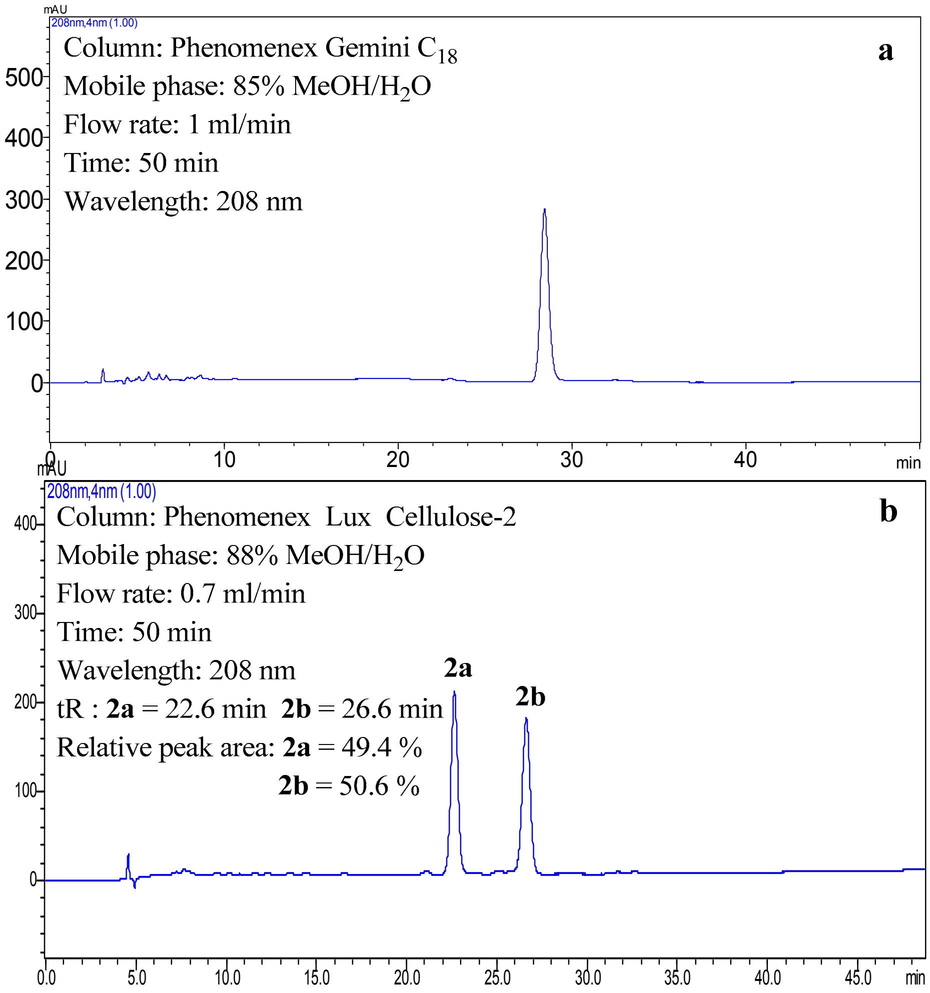

Figure S1. HPLC analysis of **2** (**a**: the analysis of 2 on routine ODS HPLC; **b**: the analysis of 2 on chiral HPLC).

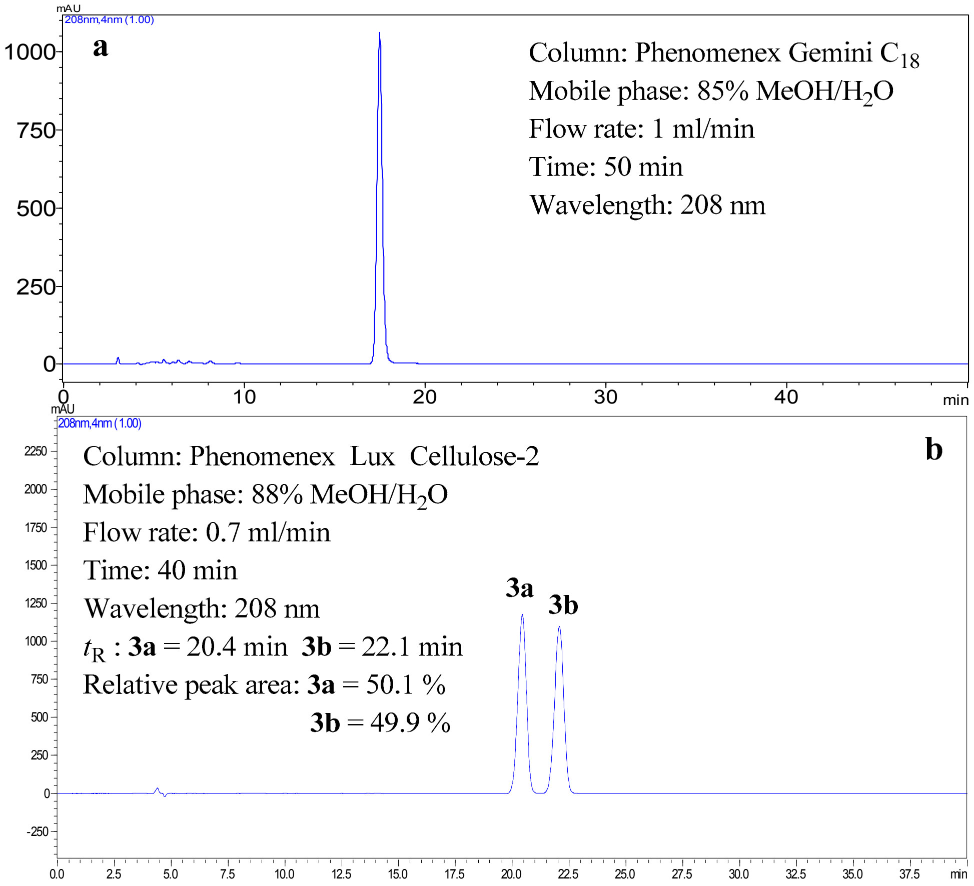

Figure S2. HPLC analysis of **3** (**a**: the analysis of **3** on routine ODS HPLC; **b**: the analysis of **3** on chiral HPLC).

Quantum Chemical ECD Calculations of 1 and 3

Table S6. Conformers distribution of (5*S*)-1 in solvated models calculations at the APFD/6-31G(d).

Conformers	Contribution %
1	27.49
2	26.71
3	15.38
4	15.35
5	4.77
6	4.68
7	2.83
8	2.79

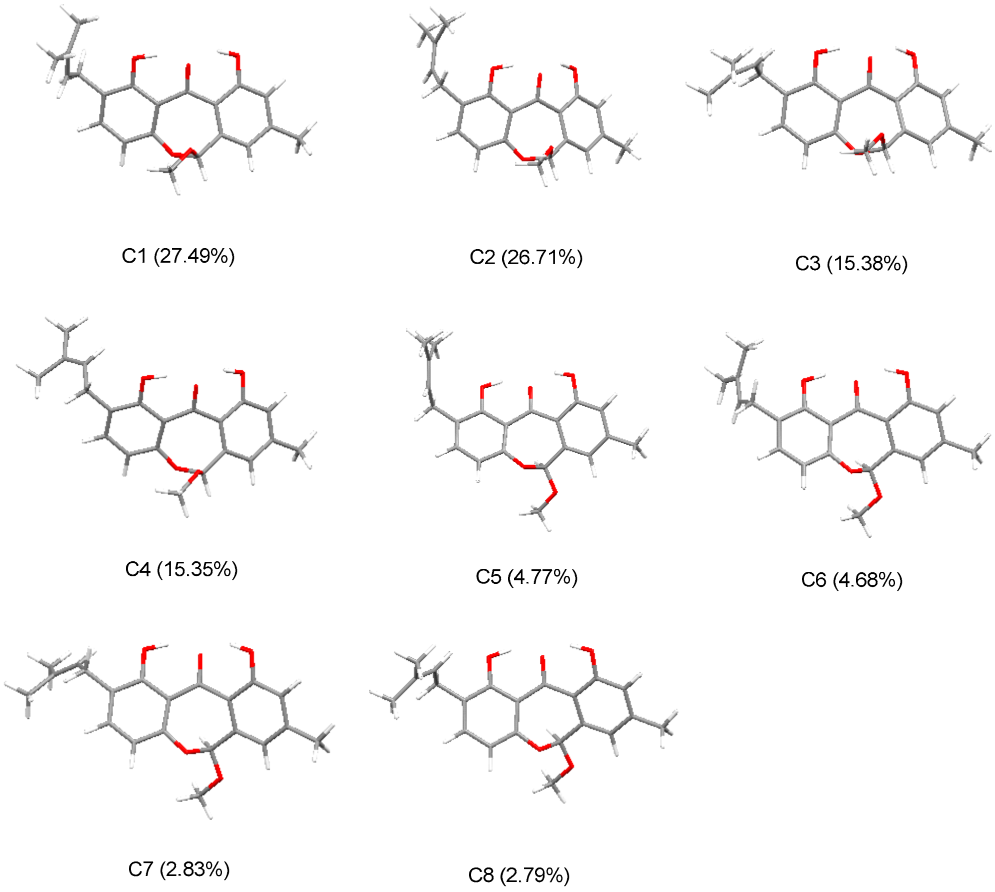

Figure S3. Most stable conformers of (5*S*)-**1**.

Table S7. Conformers distribution of (5*S*)-**3** in solvated models calculations at the APFD/6-31G(d).

Conformers	Contribution %
1	74.24
2	13.36
3	7.20
4	1.87
5	1.68
6	1.66

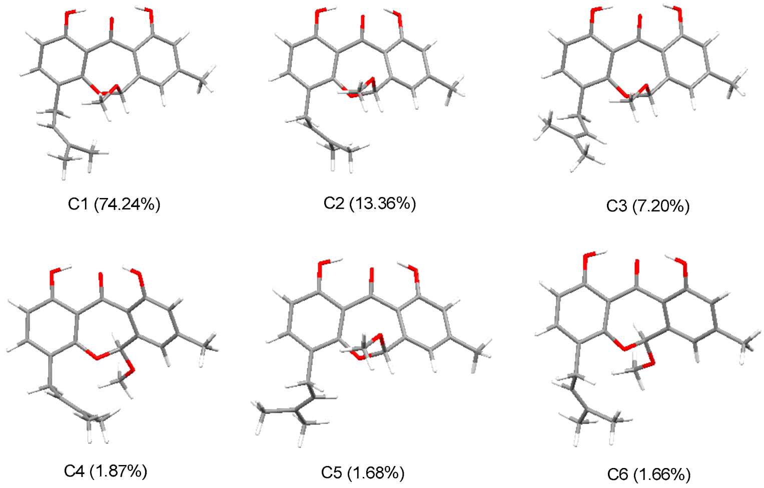

Figure S4. Most stable conformers of (5*S*)-**3**.

The 1D and 2D NMR Spectra of 1–5

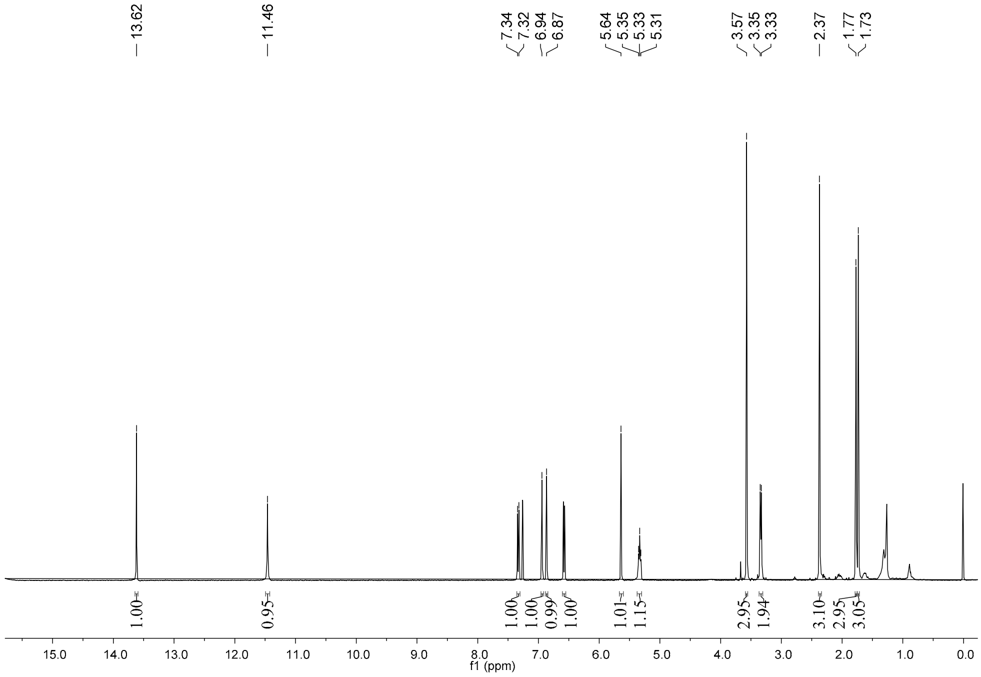

Figure S5. ^{1}H-NMR spectrum of compound **1** (400 MHz, in $CDCl_3$).

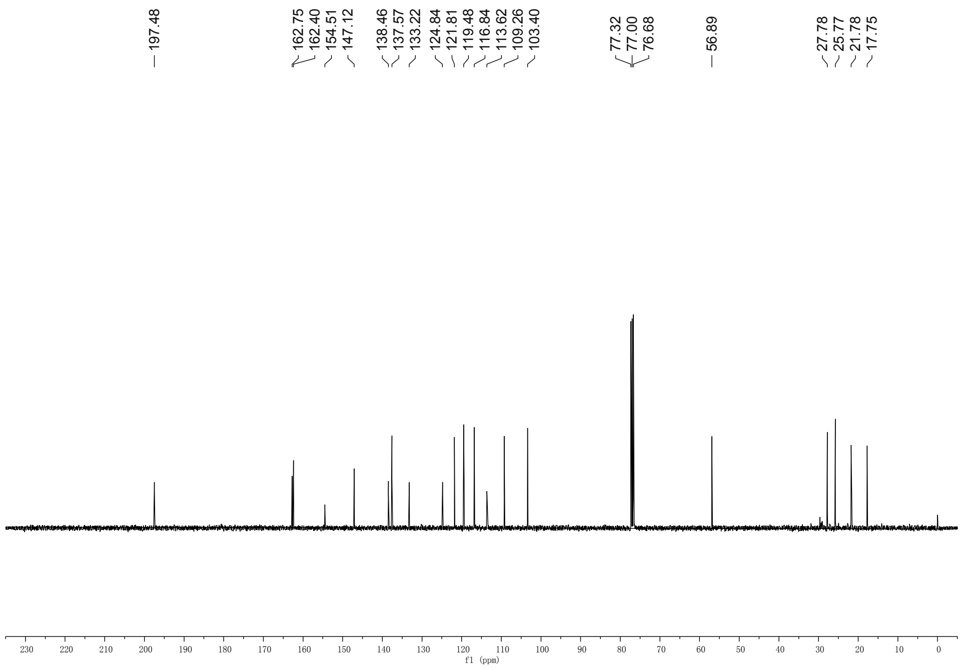

Figure S6. ^{13}C-NMR spectrum of compound **1** (100 MHz, in $CDCl_3$).

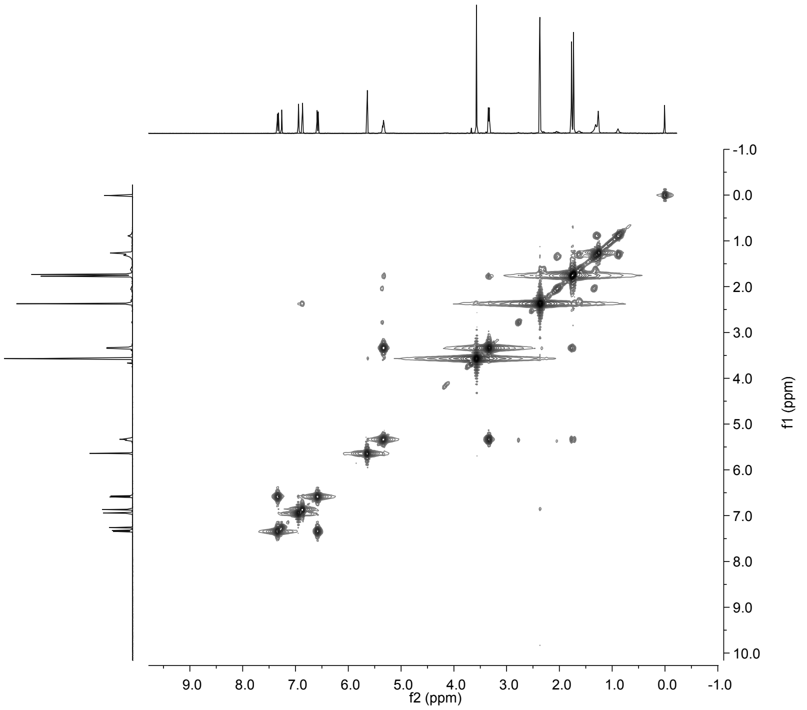

Figure S7. ^{1}H-^{1}H COSY spectrum of compound **1** (in $CDCl_3$).

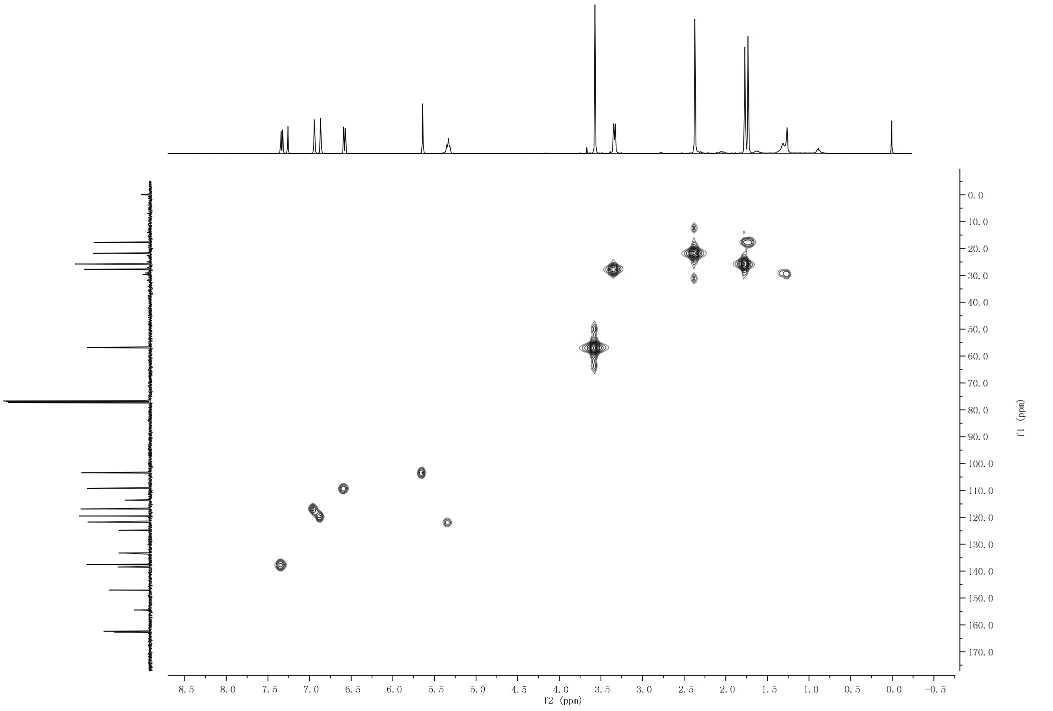

Figure S8. HSQC spectrum of compound **1** (in $CDCl_3$).

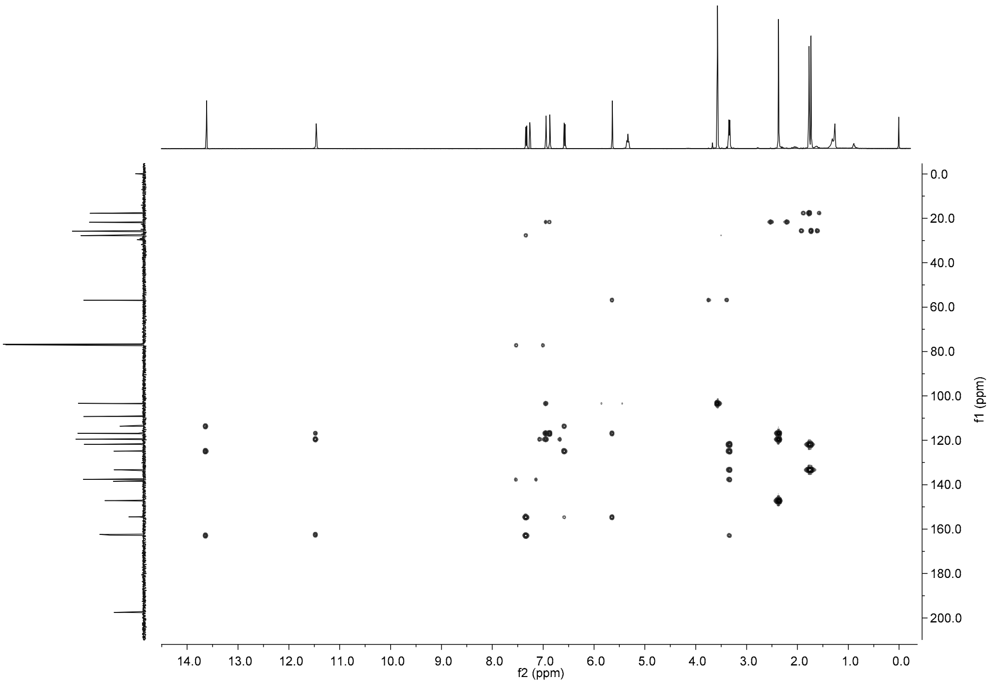

Figure S9. HMBC spectrum of compound **1** (in CDCl3).

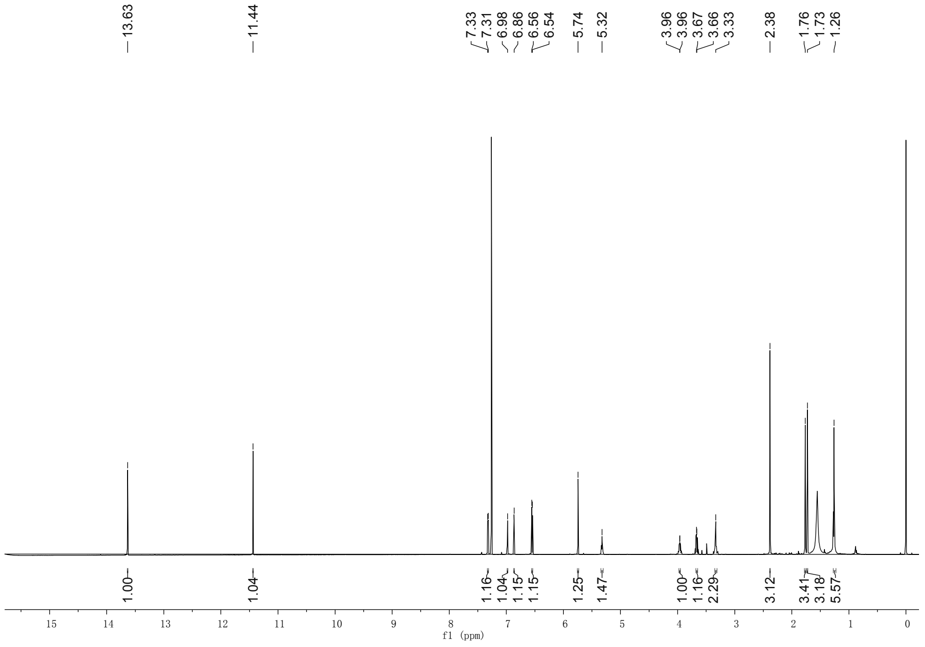

Figure S10. ^{1}H-NMR spectrum of compound **2** (600 MHz, in CDCl3).

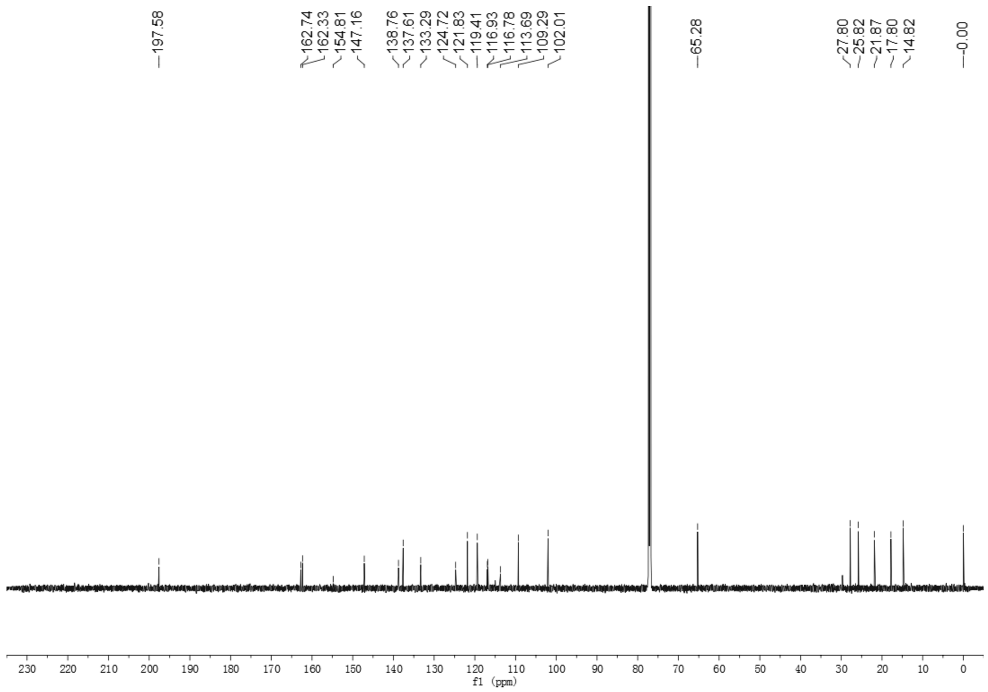

Figure S11. ^{13}C-NMR spectrum of compound **2** (150 MHz, in $CDCl_3$).

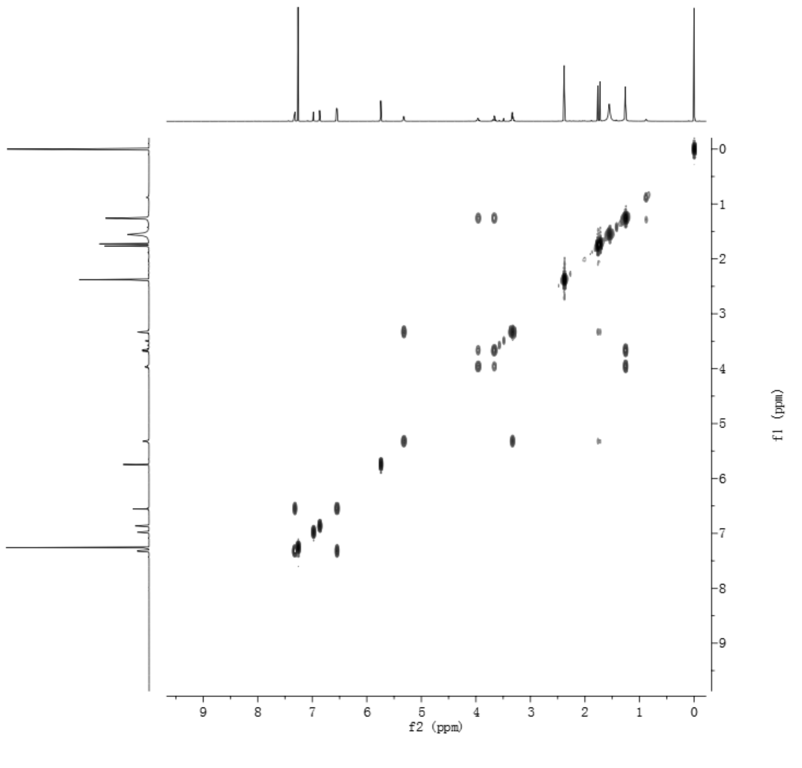

Figure S12. ^{1}H-^{1}H COSY spectrum of compound **2** (in $CDCl_3$).

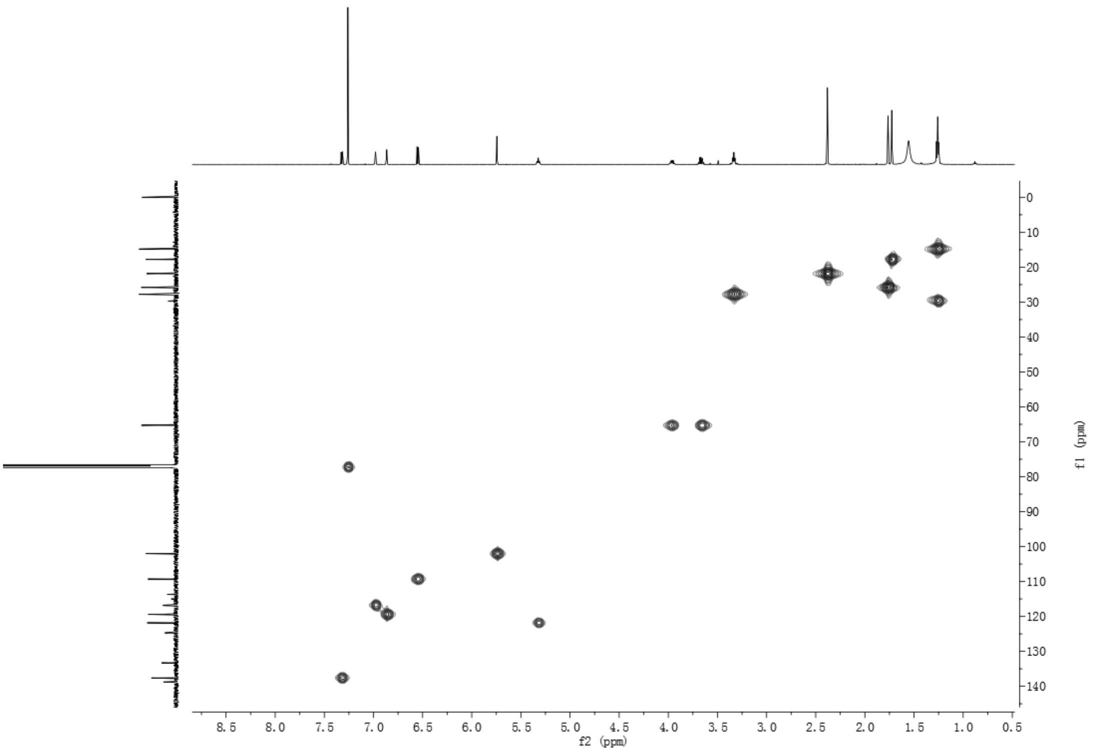

Figure S13. HSQC spectrum of compound **2** (in $CDCl_3$).

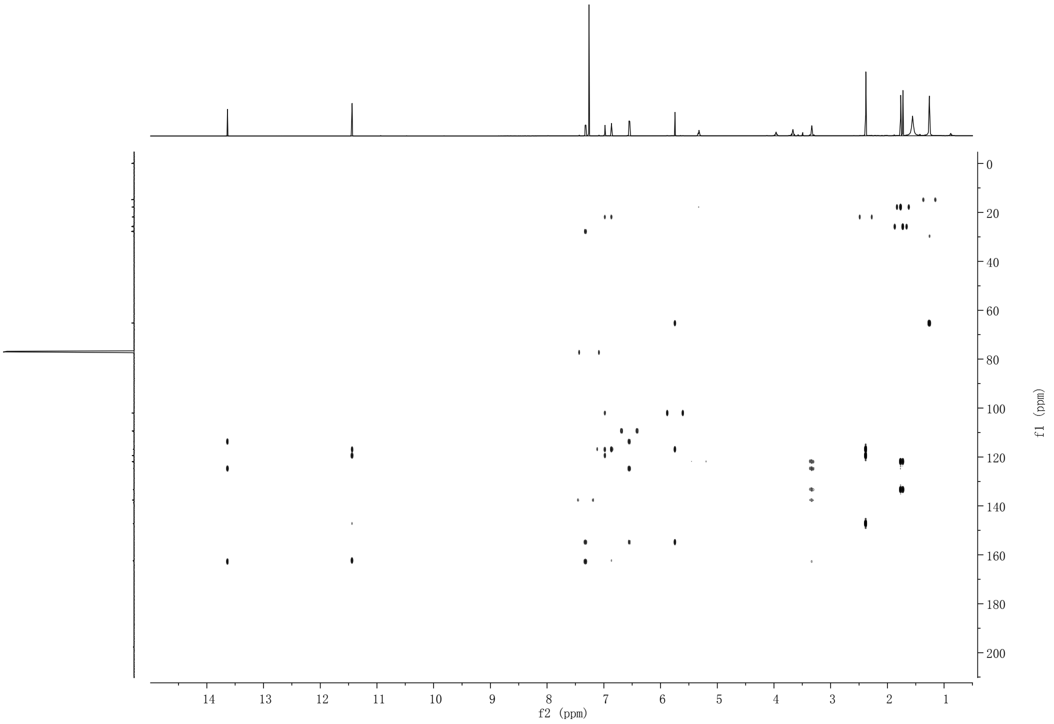

Figure S14. HMBC spectrum of compound **2** (in $CDCl_3$).

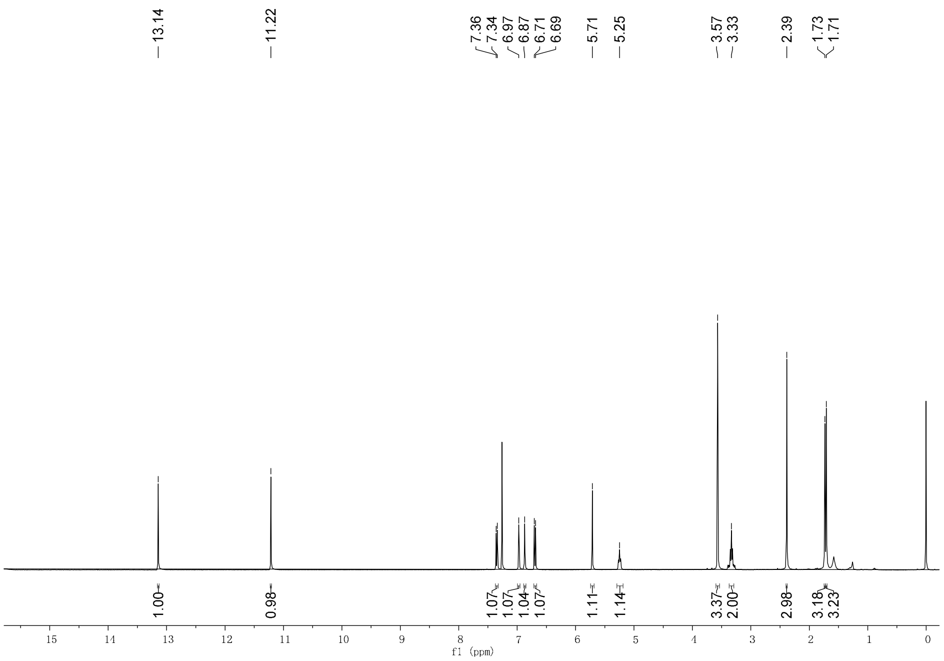

Figure S15. ^{1}H-NMR spectrum of compound **3** (400 MHz, in $CDCl_3$).

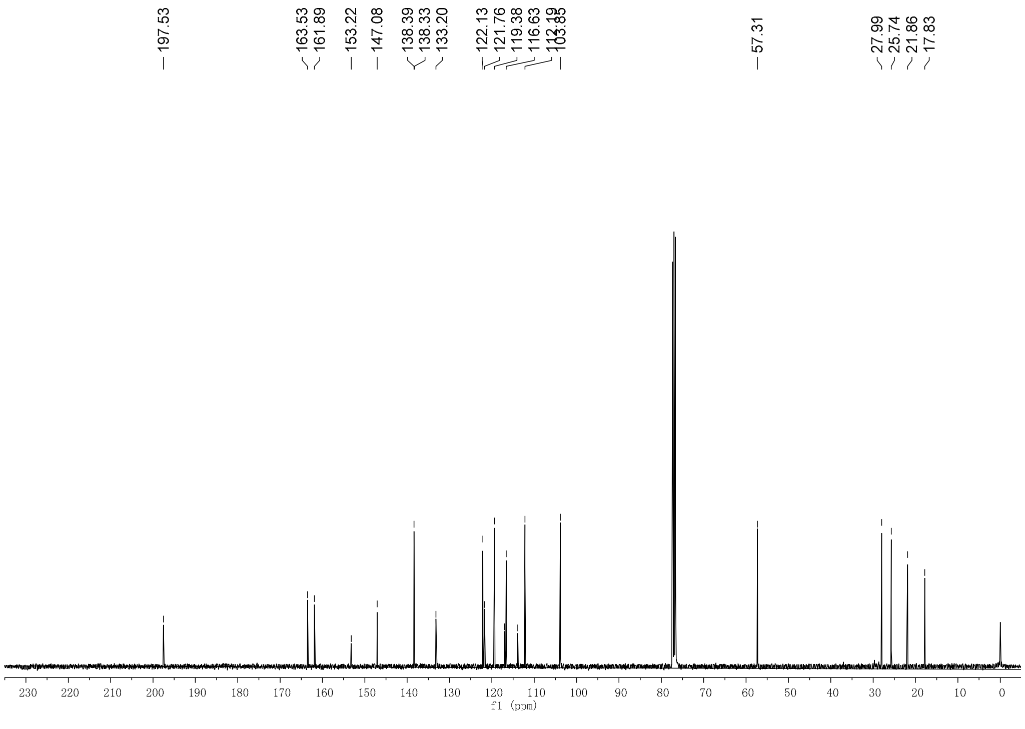

Figure S16. ^{13}C-NMR spectrum of compound **3** (100 MHz, in $CDCl_3$).

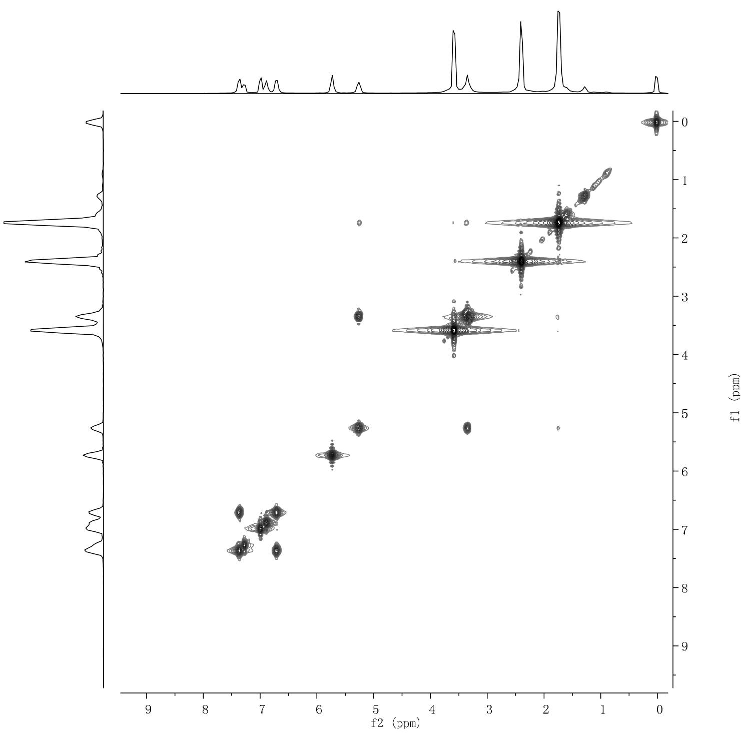

Figure S17. ^{1}H-^{1}H COSY spectrum of compound **3** (in $CDCl_3$).

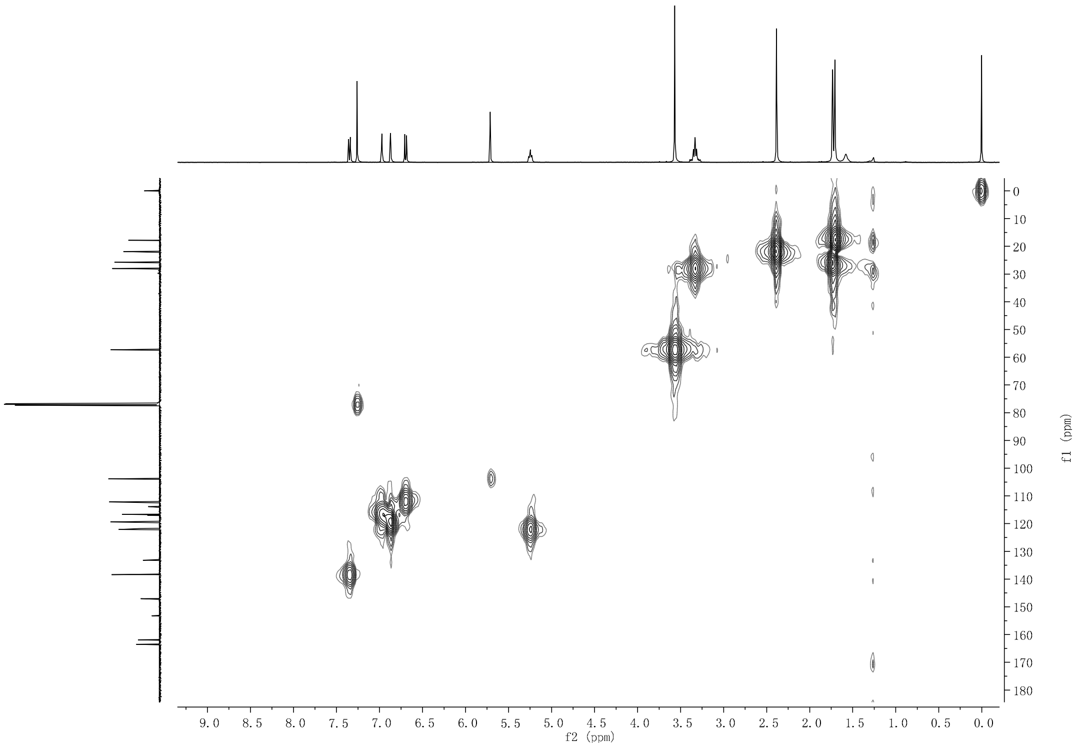

Figure S18. HSQC spectrum of compound **3** (in $CDCl_3$).

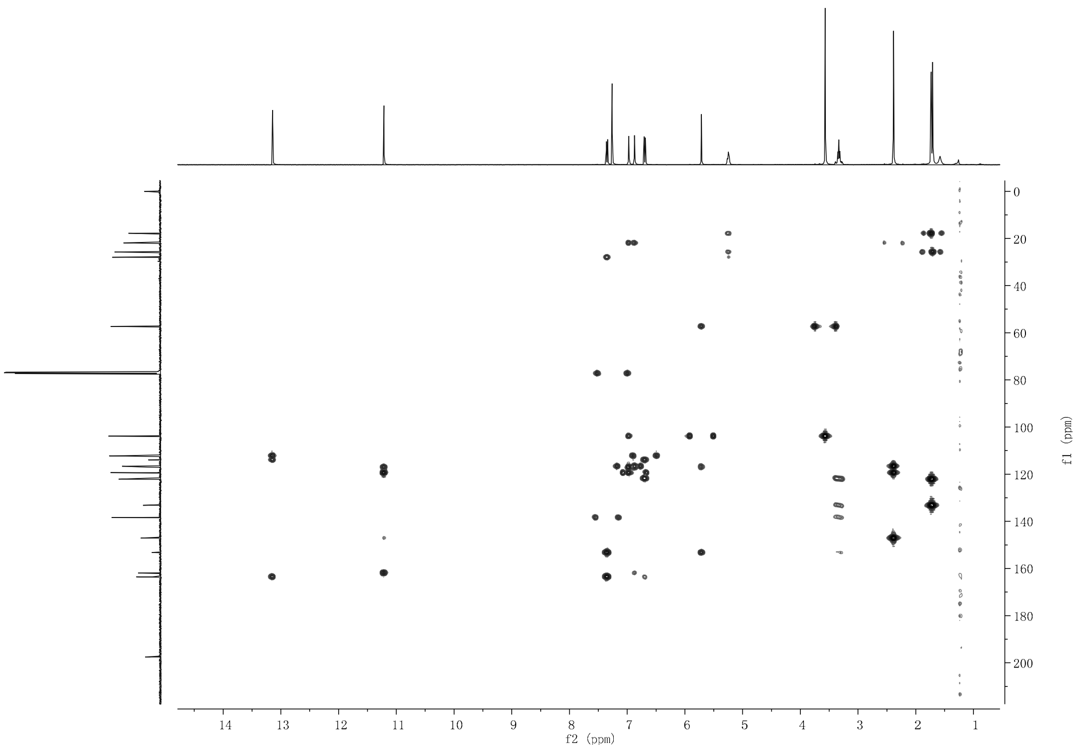

Figure S19. HMBC spectrum of compound **3** (in $CDCl_3$).

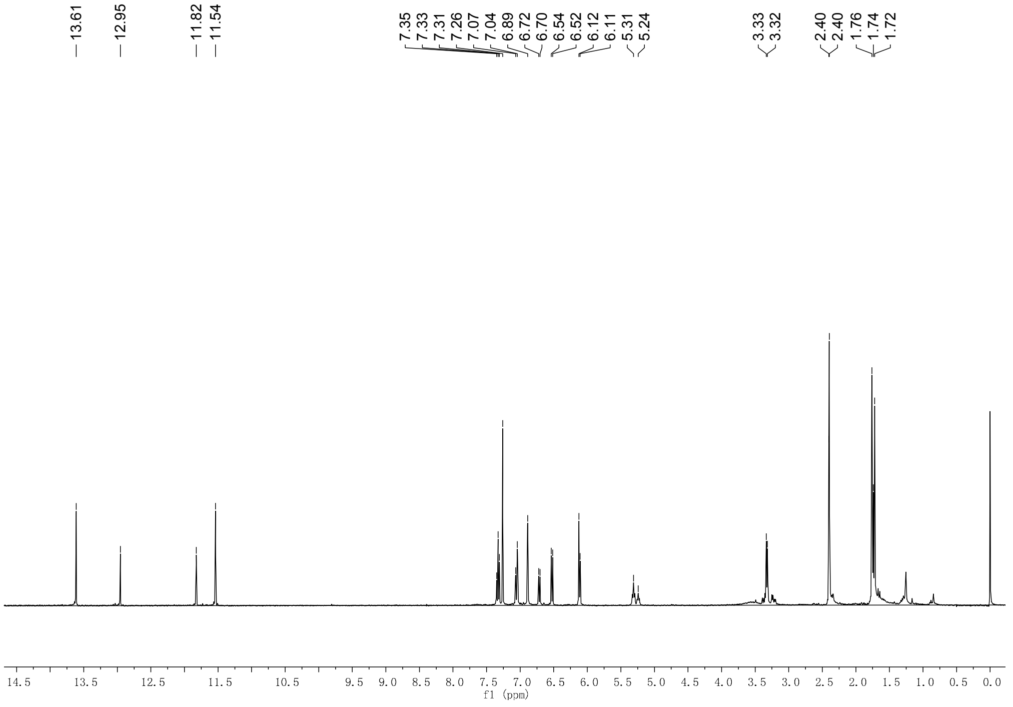

Figure S20. ^{1}H-NMR spectrum of compounds **4** and **5** (400 MHz, in $CDCl_3$).

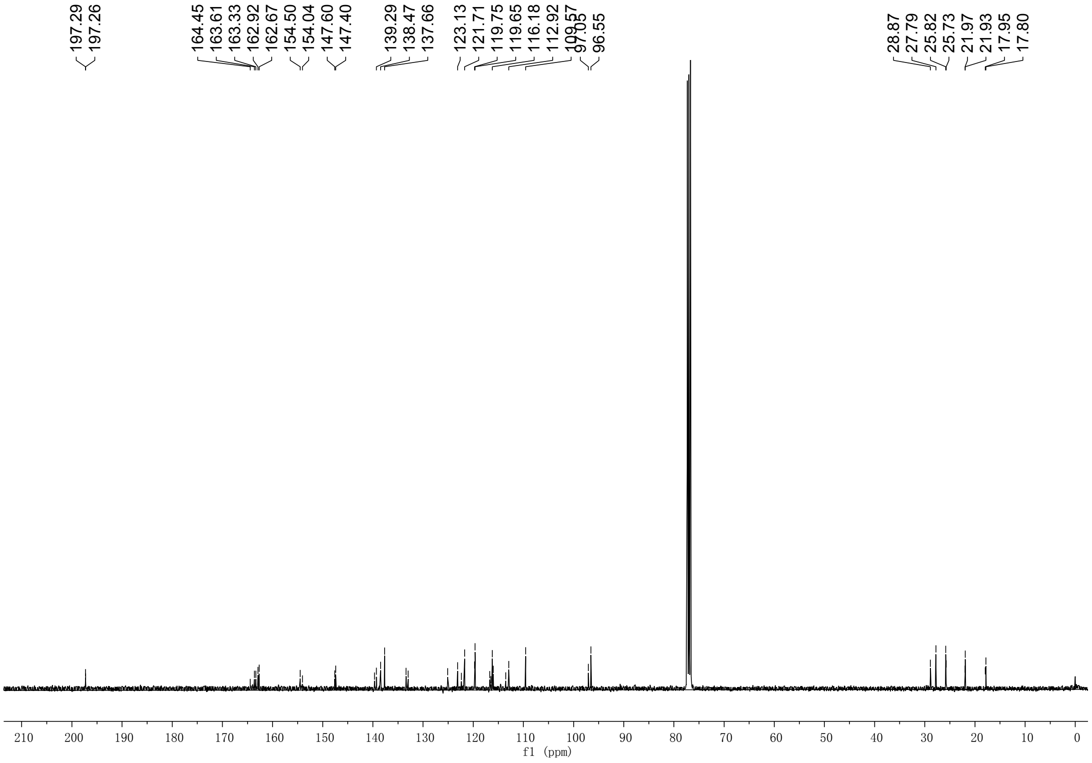

Figure S21. ^{13}C-NMR spectrum of compounds **4** and **5** (100 MHz, in $CDCl_3$).

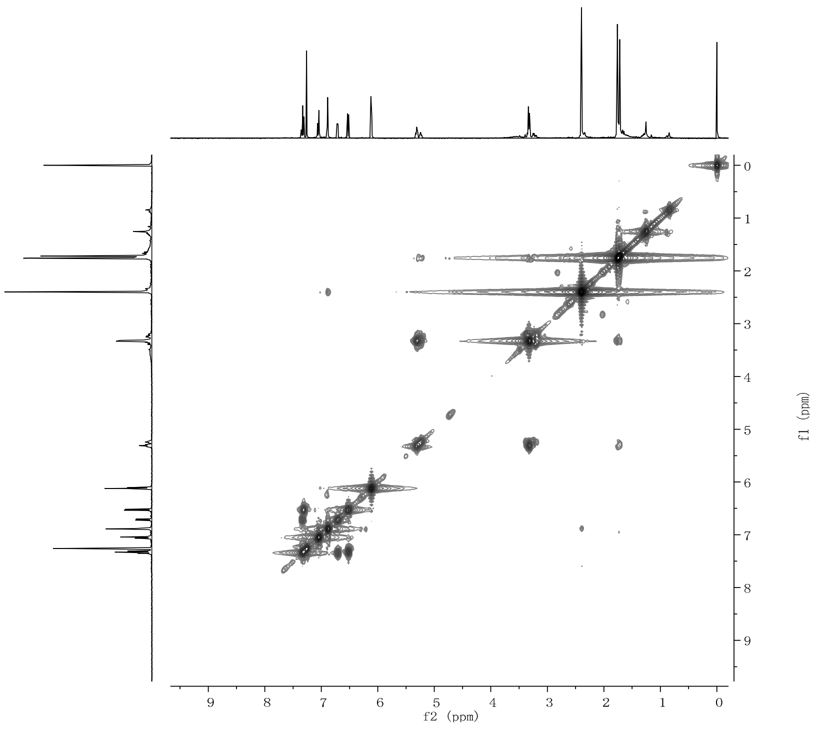

Figure S22. ^{1}H-^{1}H COSY spectrum of compounds **4** and **5** (in $CDCl_3$).

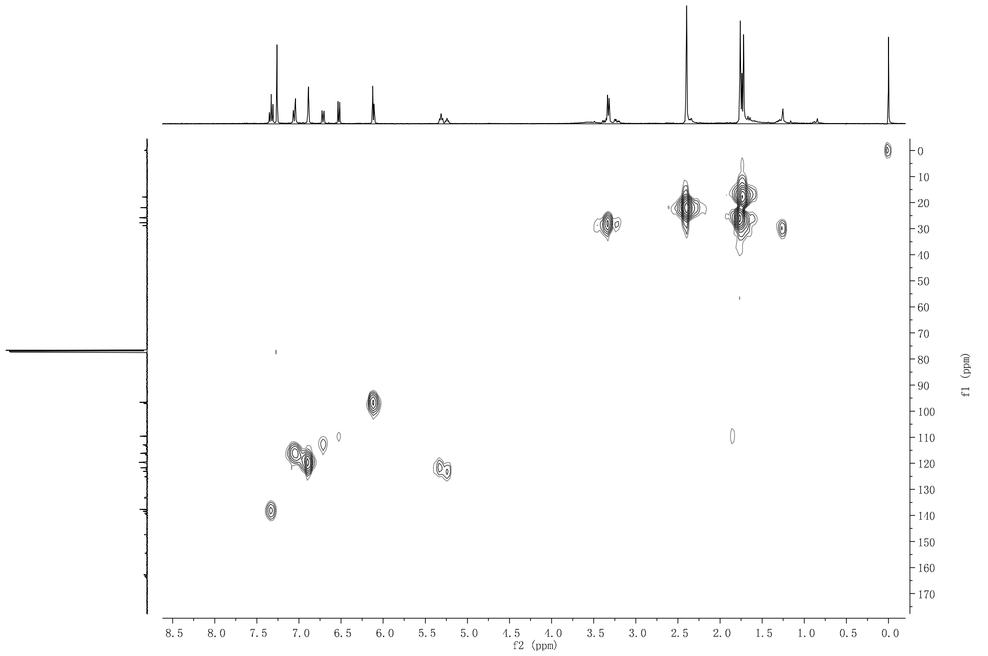

Figure S23. HSQC spectrum of compounds **4** and **5** (in $CDCl_3$).

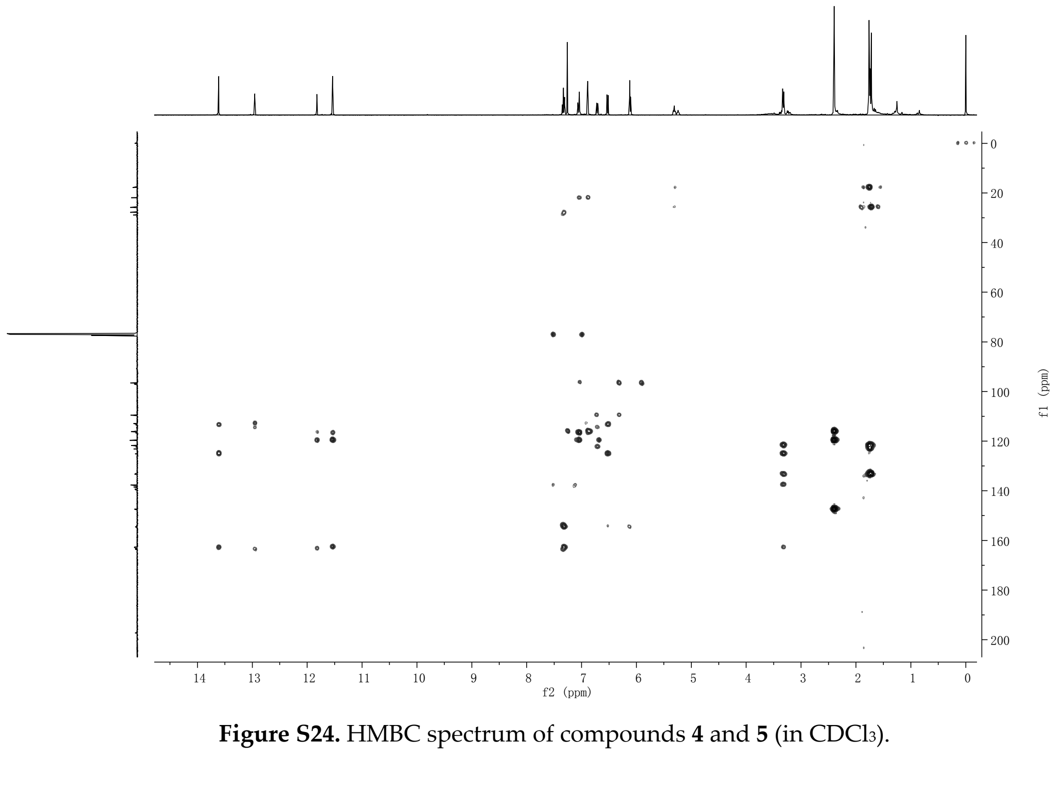

Figure S24. HMBC spectrum of compounds **4** and **5** (in $CDCl_3$).
